# Gorilla MHC class I gene and sequence variation in a comparative context

**DOI:** 10.1007/s00251-017-0974-x

**Published:** 2017-03-22

**Authors:** Jörg B. Hans, Richard A. Bergl, Linda Vigilant

**Affiliations:** 1 Max Planck Institute for Evolutionary Anthropology, Deutscher Platz 6, 04103 Leipzig, Germany; 2 North Carolina Zoological Park, Asheboro, NC 27205 USA

**Keywords:** MHC genotyping, PacBio, Next-generation sequencing, Gogo, Haplotypes, Evolution

## Abstract

Comparisons of MHC gene content and diversity among closely related species can provide insights into the evolutionary mechanisms shaping immune system variation. After chimpanzees and bonobos, gorillas are humans’ closest living relatives; but in contrast, relatively little is known about the structure and variation of gorilla MHC class I genes (*Gogo*). Here, we combined long-range amplifications and long-read sequencing technology to analyze full-length MHC class I genes in 35 gorillas. We obtained 50 full-length genomic sequences corresponding to 15 *Gogo-A* alleles, 4 *Gogo-Oko* alleles, 21 *Gogo-B* alleles, and 10 *Gogo-C* alleles including 19 novel coding region sequences. We identified two previously undetected MHC class I genes related to *Gogo-A* and *Gogo-B*, respectively, thereby illustrating the potential of this approach for efficient and highly accurate MHC genotyping. Consistent with their phylogenetic position within the hominid family, individual gorilla MHC haplotypes share characteristics with humans and chimpanzees as well as orangutans suggesting a complex history of the MHC class I genes in humans and the great apes. However, the overall MHC class I diversity appears to be low further supporting the hypothesis that gorillas might have experienced a reduction of their MHC repertoire.

**Electronic supplementary material:**

The online version of this article (doi:10.1007/s00251-017-0974-x) contains supplementary material, which is available to authorized users.

## Introduction

Molecules encoded by the MHC class I genes play an important role in the defense against intracellular pathogens (Klein and Figueroa 1986). Through the recognition and presentation of antigens, MHC class I molecules function as stimulatory ligands for lymphocytes of the adaptive and innate immune system. Cytotoxic CD8+ T cells recognize antigens that are presented by MHC class I molecules on the cell surface while some MHC class I allotypes, in addition, interact with killer cell immunoglobulin-like receptors (KIR receptors) that are primarily expressed on natural killer cells (NK cells) and regulate the inhibition and activation of NK cell responses (Parham and Ohta 1996; Lanier 2005). Due to host-pathogen interactions, the genes encoding MHC class I molecules are subject to rapid evolution (Hedrick 1994; Hughes and Hughes 1995). High-sequence polymorphism of MHC class I genes provides diversity in antigen-binding repertoires while recombination between MHC class I genes facilitates the generation of novel specificities (Ohta 1983; Jakobsen et al. 1998). In humans and other nonhuman primates, the MHC class I multigene family has diversified through recurrent duplications and deletions leading to species-specific patterns of intra- and interlocus variability (Adams and Parham 2001; Kulski et al. 2002; Kelley et al. 2005). Thus, interspecies comparisons can provide important insights into the evolution of MHC class I genes and possibly the resistance and susceptibility to infectious diseases (Sommer 2005; Shiina et al. 2006).

Sharing a common ancestor approximately 10 million years ago, gorillas are humans’ closest living relatives after chimpanzees and bonobos (Ruvolo et al. 1991; Scally et al. 2012). However, in contrast to humans and chimpanzees, surprisingly little is known about the structure and variation of MHC genes in gorillas (Hans et al. 2015). In general, it is presumed that gorillas have a MHC class I region similar to humans as concluded from the presence of gorilla orthologues (*Gogo-A*, *-B*, and *-C*) of all classical human MHC class I genes (*HLA-A*, *-B*, *-C*) (Venditti et al. 1996; Lawlor et al. 1991; Watkins et al. 1991; Adams and Parham 2001). The previous sampling of *Gogo* class I alleles derived from only four individuals showed that *Gogo-A* alleles segregate only into one of the two ancient lineages of *HLA-A*, the *A2* lineage (Lawlor et al. 1991; Adams and Parham 2001). However, among the five identified complementary DNA (cDNA) sequences related to *HLA-A*, two appeared particularly divergent (Lawlor et al. 1991; Adams and Parham 2001). The allele Gogo-A*05:01 shares remarkable sequence similarity with the human pseudogene *HLA-Y* (also designated as *HLA-BEL*, *-COQ*, and *-DEL*) and was speculated to represent an ancestral *A* locus allele predating the divergence of the gorilla and human MHC (Lawlor et al. 1991; Williams et al. 1999; Coquillard et al. 2004; Marsh et al. 2010). The *Gogo-A3* or *-Oko* allele, however, shares sequence homology with the human pseudogene *H* and other classical *A* locus alleles but until recently, it was unclear whether *Gogo-Oko* is a divergent *A* locus allele or a separate gene (Lawlor et al. 1991; Watkins et al. 1991; Adams and Parham 2001). Comparative analysis of a single gorilla MHC haplotype showed that *Gogo-Oko* is syntenic to the chimpanzee *A*-related locus *Patr-AL* (Gleimer et al. 2011). *Patr-AL* is present on only half of the surveyed chimpanzee MHC haplotypes and is in strong linkage disequilibrium with *Patr-A*, the chimpanzee orthologue of *HLA-A* (Adams et al. 2001; Geller et al. 2002). In contrast, the gorilla MHC haplotype with *Gogo-Oko* lacks the genomic block containing the *A* locus (Gleimer et al. 2011). Thus, gorillas possess an additional and presumably functional *A*-related locus which is characterized by an unusual haplotype structure but little is known about its frequency and variation (Lawlor et al. 1991; Watkins et al. 1991). Nevertheless, that certain *Gogo-A* alleles are orthologous to *HLA-A* suggests that some gorilla MHC haplotypes have retained the genomic block with the *A* locus (Gleimer et al. 2011). In sum, there are indications for a complex history of the *A* and *A*-related genes in gorillas and their closest relatives.

In contrast, it is presumed that, similar to humans and chimpanzees, gorillas possess a single *MHC-B* and *MHC-C* gene, respectively, whereas orangutan haplotypes have multiple copies of *MHC-B* and some lack the *MHC-C* (Chen et al. 1992; Adams et al. 1999; Adams and Parham 2001; de Groot et al. 2016). For *Gogo-B*, the gorilla orthologue of *HLA-B*, 11 alleles have been characterized among 18 individuals which implies that *Gogo-B* is, like its human and chimpanzee equivalent, the most polymorphic of the gorilla MHC class I genes (Lawlor et al. 1991; Urvater et al. 2001; Martínez-Laso et al. 2006; Abi-Rached et al. 2011). Therefore, analysis of additional gorillas will likely increase the number of alleles identified at this locus. As with *MHC-B*, it appears that the *MHC-C* gene is present on all MHC haplotypes in humans and the African great apes and accordingly, a total of seven *Gogo-C* alleles have been identified among seven individuals (Lawlor et al. 1991; Urvater et al. 2001). However, to gain a more comprehensive understanding of the gorilla MHC class I diversity and the evolution of these loci in great apes and humans, characterization of more individuals is needed.

Despite their importance, MHC class I genes are rarely characterized comprehensively which at least in part is due to the technical difficulty of unambiguously determining the sequences of both alleles using conventional sequencing approaches. Indeed, the majority of *Gogo* class I alleles have been described from cDNA sequences. Emerging long-read sequencing technologies have the ability to reliably characterize full-length MHC class I genes, as has recently been shown in humans (Mayor et al. 2015). Similarly, we here applied long-range PCR (LR-PCR) to amplify full-length gorilla MHC class I genes from high-quality genomic DNA comprising complete coding region sequences and intervening introns. This approach has several advantages. First, sequencing of polymorphism within non-coding regions increases the level of detail at which an allele is characterized. Thus, the higher resolution enables the recognition of heterozygous genotypes if individual alleles are identical in coding region sequences. Second, amplification from genomic DNA allows the characterization of low- or non-expressed alleles which are undetectable at the cDNA level. Third, LR-PCR primers can be placed in conserved regions outside the genes thereby allowing the characterization of the entire allelic variation. Here, we use this approach to characterize 35 gorillas including a large number of wild-born individuals to achieve a more comprehensive description of MHC class I variation in gorillas with comparison to data from human and chimpanzees.

## Material and methods

### Sample collection and DNA extraction

Taxonomically, gorillas are classified into two species: the western gorilla (*Gorilla gorilla*) and the eastern gorilla (*Gorilla beringei*), each of which have been further subdivided into two subspecies. The western species is comprised of western lowland gorillas (*Gorilla gorilla gorilla*) and Cross River gorillas (*Gorilla gorilla diehli*) while mountain gorillas (*Gorilla beringei beringei*) and eastern lowland gorillas (*Gorilla beringei graueri*) comprise the eastern species (Groves 2001). Our study consisted of samples from 35 captive individuals including one eastern lowland gorilla, one Cross River gorilla, and 33 western lowland gorillas. As our aim was to assess MHC class I gene variation among natural populations, our sampling focused on wild-born individuals (*n* = 25) as well as captive-born individuals of the first and second generation with at least one wild-born parent (*n* = 9) (Online Resources 1 and 2). Based on reported capture locations, we could broadly infer geographical origins of most wild-born gorillas (Online Resource 1). Genomic DNA was extracted from 17 whole blood, 1 buffy coat, and 5 tissue samples using either the Gentra Puregene Blood Kit (QIAGEN, Hilden, Germany) or the DNeasy Blood & Tissue Kit (QIAGEN) according to the manufacturer’s instructions. In addition, 11 pre-extracted whole blood samples collected as part of a previous study were purified using the QIAamp DNA Mini Kit (QIAGEN) (see for details Thalmann et al. 2007). Furthermore, commercially available genomic DNA from the gorilla cell line “EB (JC)” was purchased from the European Collection of Cell Cultures (ECACC) (Public Health England, Porton Down, Salisbury, UK) (Online Resource 1). Quantities of DNA templates were estimated through spectrophotometric measurements using a NanoDrop ND-1000 (Thermo Scientific, Waltham, MA, USA) and concentrations were adjusted to approximately 25 ng/μL for the LR-PCR amplifications. Collection of samples was conducted under the supervision of ethical committees and, if required, CITES permissions (Convention on International Trade in Endangered Species of Wild Fauna and Flora) were obtained.

### Primer design, LR-PCR, and sequencing of MHC class I genes

To design gene-specific primers, we analyzed all available MHC class I gene sequences from the gorilla and its closest relatives, human, chimpanzee, and bonobo. Following the construction of multiple alignments using ClustalW as implemented in BioEdit version 7.2.0 (Hall 1999), primers were manually placed in interspecies conserved regions and where necessary, degenerate bases were included to ensure the amplification of the complete allelic variation of each gene. Following the equation given in SantaLucia (1998), primers were chosen to have melting temperatures of at least 64 °C to enhance reaction specificity. Finally, primers were checked in silico for potential self-complementary and hairpin formation using the program OligoCalc (Kibbe 2007). Primers were designed to encompass entire coding sequences with the forward primer located in the promoter-enhancer region and the reverse primer located in the 3′-untranslated region. Due to high sequence similarity outside of the coding regions, a universal primer pair was designed to nonspecifically amplify both the gorilla *MHC-A* and *-Oko* genes. Sequences of primers used in the present study are given in Online Resource 3.

Individual LR-PCRs (gorilla *MHC-A/Oko*, *-B*, and *-C*) were performed in a final volume of 50 μL consisting of 1× Crimson LongAmp Taq Reaction Buffer, 100 μM dNTPs, 0.2 μM of each primer, 0.1 unit Crimson LongAmp Taq Polymerase (New England Biolabs, Frankfurt am Main, Germany), and 2 μL of DNA template. PCR amplifications consisted of a two-step PCR program with an initial denaturation at 94 °C for 2 min, followed by 40 cycles of 94 °C for 20 s and 68 °C for 9 min and a final extension at 68 °C for 15 min. After confirmation of successful amplification by gel electrophoresis, amplicons were individually purified using 1.0× ratio of Agencourt AMPure XP beads (Beckman Coulter, Krefeld, Germany) according to the manufacturer’s instructions, and concentrations were measured with a NanoDrop ND-1000 spectrophotometer (Thermo Scientific). The three amplicons of each individual were pooled in equimolar ratios and used to construct single-molecule real-time (SMRT) cell libraries using PacBio barcoded adapters for multiplex sequencing following the manufacturer’s protocol (Pacific Biosciences, Menlo Park, CA, USA). After blunt-end ligation of a symmetric 16-bp barcoded adapter, up to 10 uniquely barcoded amplicon pools were multiplexed prior to two DNA damage repair steps and exonuclease treatment. The quantity and quality of each library was checked using an Agilent DNA 7500 chip and the 2100 Bionanalyzer (Agilent Technologies, Santa Clara, CA, USA). The DNA/Polymerase Binding kit P6 v2 (Pacific Biosciences) was used to bind polymerases to templates followed by using the MagBead kit (Pacific Biosciences) for uniform loading of SMRT cells. Libraries with a concentration of 40 pM were sequenced on the PacBio RS II instrument (Pacific Biosciences) using the C4 chemistry, stage start, and a movie collection time of 240 min. A total of nine SMRT cells were sequenced yielding 654,289 polymerase reads (mean per SMRT cell 79,464 ± 14,220). The long amplicon analysis (LAA) as implemented in the SMRT Analysis (version 2.3.0) was used to determine phased consensus sequences followed by manual trimming of primer sequences. To resolve individual allele ambiguities between replicates, we applied the minor variant analysis implemented in the same software package.

### Genotype estimation and validation

As detailed below, we validated individual MHC genotypes through (i) the comparison of consensus sequences obtained from two independent amplifications; (ii) the analysis of four individuals with completely or partially known genotypes from previous studies; and (iii) the analysis of allelic inheritance patterns among related individuals, i.e., one parent-offspring trio and one father-offspring dyad. Full-length genomic coding sequences were submitted to the MHC-IPD NHP database and given official allele designations by the nonhuman primate MHC nomenclature committee (Maccari et al. 2017). In this context, the MHC of a species is abbreviated by a four-letter code which corresponds to its scientific name. Specifically, the MHC of the western gorilla (*G. gorilla*) is defined by *Gogo*, whereas *Gobe* refers to the MHC of the eastern gorilla (*G. beringei*) (Klein et al. 1990; de Groot et al. 2012). As listed in Table 1, the majority of alleles identified in the eastern lowland gorilla were also found in western gorillas. However, please note that allele designations differ between the two gorilla species.

**Table 1:** Gorilla MHC class I alleles identified in the present study and their respective GenBank accession numbers

Allele	Gorilla species/subspecies	Number of individuals	Accession number
Gogo-A*01:01:01	*G. g. gorilla / diehli*	4 / 1	KY189923
Gogo-A*01:01:02	*G. g. gorilla*	3	KY189924
Gogo-A*04:01:01:01	*G. g. gorilla*	17	KY189925
Gogo-A*04:01:01:02	*G. g. gorilla*	1	KY189926
Gogo-A*04:01:01:03	*G. g. gorilla*	5	KY189927
Gogo-A*04:01:01:04	*G. g. gorilla*	1	KY189928
Gogo-A*04:01:01:05	*G. g. gorilla*	10	KY189929
Gogo-A*07:01:01:01	*G. g. gorilla*	2	KY189930
Gogo-A*07:01:01:02	*G. g. gorilla*	1	KY189931
Gogo-Oko*01:01	*G. g. gorilla*	9	KY189932
Gogo-Oko*01:02	*G. g. gorilla*	2	KY189933
Gobe-Oko*01:01	*G. b. graueri*	1	KY189933
Gogo-Oko*02:01	*G. g. gorilla*	2	KY189934
Gogo-Oko*02:02	*G. g. gorilla / diehli*	1 / 1	KY189935
Gogo-A*05:02:01:01 N	*G. g. gorilla*	12	KY189936
Gogo-A*05:02:01:02 N	*G. g. gorilla / diehli*	11 / 1	KY189937
Gogo-A*05:03:01:01 N	*G. g. gorilla*	6	KY189938
Gogo-A*05:03:01:02 N	*G. g. gorilla*	6	KY189939
Gogo-A*05:03:01:03 N	*G. g. gorilla*	5	KY189940
Gogo-A*05:04 N	*G. g. gorilla*	2	KY189941
Gogo-B*01:01:01:01	*G. g. gorilla / diehli*	12 / 1	KY189942
Gogo-B*01:01:01:02	*G. g. gorilla*	2	KY189943
Gogo-B*01:02	*G. g. gorilla*	1	KY189944
Gogo-B*01:03	*G. g. gorilla*	8	KY189945
Gogo-B*01:04:01:01	*G. g. gorilla*	2	KY189946
Gogo-B*01:04:01:02	*G. g. gorilla*	1	KY189947
Gogo-B*02:01	*G. g. gorilla*	5	KY189948
Gogo-B*03:01	*G. g. gorilla*	6	KY189949
Gogo-B*03:02	*G. G. diehli*	1	KY189950
Gogo-B*03:03	*G. g. gorilla*	2	KY189951
Gogo-B*04:01:01:01	*G. g. gorilla*	1	KY189952
Gogo-B*04:01:01:02	*G. g. gorilla*	9	KY189953
Gogo-B*05:01	*G. g. gorilla*	3	KY189954
Gogo-B*05:02	*G. g. gorilla*	5	KY189955
Gogo-B*06:01	*G. g. gorilla*	2	KY189956
Gogo-B*07:01	*G. g. gorilla*	4	KY189957
Gogo-B*07:02	*G. g. gorilla*	2	KY189958
Gogo-B*07:03	*G. g. gorilla*	1	KY189959
Gogo-B*07:04	*G. g. gorilla*	1	KY189960
Gogo-B*12:01	*G. g. gorilla*	1	KY189961
Gobe-B*13:01	*G. b. graueri*	1	KY189962
Gogo-C*01:01:01	*G. g. gorilla*	11	KY189963
Gogo-C*01:01:02	*G. g. gorilla*	11	KY189964
Gogo-C*01:01:03	*G. g. diehli*	1	KY189965
Gogo-C*01:03	*G. g. gorilla*	1	KY189966
Gogo-C*01:04	*G. g. gorilla*	1	KY189967
Gogo-C*02:02	*G. g. gorilla / diehli*	4 / 1	KY189968
Gobe-C*02:01	*G. b. graueri*	1	KY189968
Gogo-C*02:03	*G. g. gorilla*	11	KY189969
Gogo-C*02:04:01:01	*G. g. gorilla*	2	KY189970
Gogo-C*02:04:01:02	*G. g. gorilla*	1	KY189971
Gogo-C*02:05	*G. g. gorilla*	2	KY189972

Alleles with novel coding region sequences are highlighted in bold

As mentioned above, LR-PCR amplifications and sequencing were performed twice for each gorilla sample to validate generated sequences. However, after consensus clustering of barcoded subreads, 5 of 35 samples showed low coverage for gorilla *Gogo-A/Oko* amplicons in both replicates, probably due to unequal pooling of LR-PCR products and thus were repeated. The total number of subreads per sample and replicate ranged from 1016 to 3172, with an average of 2165 ± 518. More specifically, total numbers of subreads per sample and replicate ranged from 264 to 1414 (mean 801 ± 273) for the gorilla *Gogo-A/Oko* amplicons, from 206 to 1464 (751 ± 278) for the *Gogo-B* amplicons, and from 500 to 1000 (613 ± 198) for the *Gogo-C* amplicons. Subread coverage of consensus sequences, which by default cannot exceed 500, was on average 270 ± 158 (range 25–500), 370 ± 138 (51–500), and 447 ± 97 (225–500) for *Gogo-A/Oko*, *-B*, and *-C*, respectively (ConsensusTools v2.3.0 Documentation 2014). The mean expected accuracy of consensus sequences based on the consensus-calling algorithm Quiver was 99.958% (±0.102%, range 98.132–99.994%).

Overall, 424 of 446 high-quality consensus sequences were completely identical between the two LR-PCR reactions. The remaining 22 sequences differed in homopolymer stretches or had highly similar alleles but the variants could be resolved. In detail, 16 *Gogo-B* and *-Oko* consensus sequences obtained among the replicates of seven samples were nearly identical except for inconsistencies in homopolymer regions, a common issue of most sequencing technologies (Mayor et al. 2015). Although it is unclear whether these inaccuracies reflect PCR-mediated errors due to “polymerase slippage” or are a result of the SMRT DNA sequencing technology, the affected bases were located in the 3′ untranslated region and hence had no impact on further analyses (but see Ross et al. 2013). The remaining six inconsistent sequences were observed among the replicates of two samples and consisted of three highly similar *Gogo-B* consensus sequences, each of which differed from one another at a single base-pair position. Regarding the first sample (“Carlos”), a deletion was observed in the consensus sequence of the first replicate whereas consensus sequences of the second replicate showed two different bases at that specific position. Translation of the consensus sequence with the deletion resulted in a frameshift and premature termination. In contrast, the consensus sequences with different bases translated into two different amino acid sequences and identical coding sequences containing each of the two base variants were found in other samples, including its offspring “Kwan”, thus indicating two alleles. Similarly, consensus sequences of the second sample (“Binti Jua”) differed at one position in an intronic region, with a deletion observed in the consensus sequence of the first replicate and consensus sequences of the second replicate containing two base variants. Only sequences containing each of the two bases were observed in other samples, thus indicating again two different alleles. These inaccuracies probably occurred as a result of the high homogeneity shared between the two sequences (personal communication, Philip Lobb from Pacific Biosciences). If there is an insufficient number of reads, consensus and variant calling performed by the LAA cannot differentiate between the two alleles to segregate reads into multiple haplotypes (ConsensusTools v2.3.0 Documentation 2014). However, to further resolve discordant alleles between replicates of these two samples, we used a reference-based analysis method which enables the detection and quantification of minor variants in a heterogeneous sample. The minor variant analysis verified the presence of two alleles differing by a single base-pair in each of the replicates of the two samples, respectively. Moreover, we did not detect pronounced deviations between replicates from both the frequency of each variant (allele frequencies in first and second replicate of “Carlos”: 0.439/0.561 and 0.419/0.581; and “Binti Jua”: 0.319/0.681 and 0.310/0.690) and its respective depth of coverage (allele coverage in first and second replicate of “Carlos”: 395/504 and 392/544; and “Binti Jua”: 696/1480 and 689/1538) which demonstrates that these inaccuracies are not due to unequal amplification efficiencies and/or allelic dropout but are a result of the LAA. However, we did not detect any differences between replicates of the other samples. Overall, we therefore conclude that obtained consensus sequences are highly reproducible and therefore accurately reflect individual MHC class I sequences.

To further validate our approach, we included three gorilla individuals with previously described MHC class I genotypes: “Banga”, Gogo-A*04:01, -B*01:01, -B*01:03, -C*01:01, and -C*02:01 (Lawlor et al. 1991); “Oko”, *Gogo-Oko*, -B*02:01, and -C*02:02 (Lawlor et al. 1991); “Beta”, Gogo-A*04:01, -A*05:01, -B*01:01, -B*03:01, -C*01:01, and -C*01:03 (Urvater et al. 2001); as well as individuals with the following full-length genomic *Gogo-B* alleles: “Beta”, Gogo-B*07:01; “Oko”, Gogo-B*02:01; and “Machi”, Gogo-B*04:01 (Abi-Rached et al. 2011). Comparison of our generated sequences to previously described cDNAs or full-length genomic sequences revealed complete identity, as summarized in Table 2. However, for the gorilla “Banga” we identified the allele Gogo-C*02:03 which was previously mischaracterized as Gogo-C*02:01 (see below).

**Table 2:**
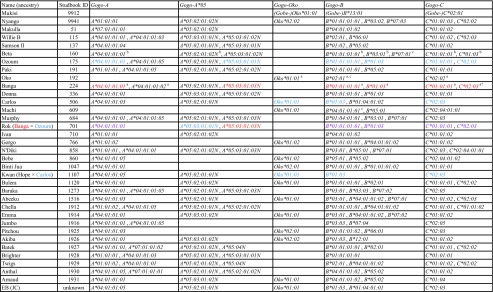
Summary of gorilla MHC class I genotypes. Individual alleles assigned with superscript numbers were completely or partially identified in previous studies. Ancestries of related individuals are shown: Red alleles are transmitted by the mother, blue alleles are transmitted by the father and purple alleles could have been transmitted by either one

^a^Lawlor et al. (1991); ^a?^ was previously mischaracterized (see text for details)

^b^Urvater et al. (2001); *Gogo-A* alleles were inferred through partial characterization of cDNA sequences (exons 2 and 3);

^c^Abi-Rached et al. (2011) characterized certain full-length genomic *Gogo-B* alleles

As a third validation step, we confirmed sharing of identical alleles within one parent-offspring trio and one father-offspring dyad (Table 2). Due to the analysis of one parent-offspring trio which included Banga’s offspring “Rok” and its sire “Ozoum”, we were able to confirm the Gogo-C*02:03 allele for “Banga” (Table 2). Taken together, we are confident that our LR-PCR approach in combination with the SMRT sequencing technology enables the reliable estimation of the entire MHC class I gene variation in gorillas.

### Phylogenetic analyses

Phylogenetic analyses were performed using full-length coding region sequences of human, chimpanzee, bonobo, gorilla, and orangutan selected to represent the range of MHC class I variation in each species. Sequences were aligned using MAFFT (Katoh et al. 2002) and phylogenies were reconstructed using the neighbor-joining (NJ) method with the Tamura-Nei model and 500 bootstrap replicates as implemented in MEGA version 6 (Tamura et al. 2013). However, inference of orthologous relationships between MHC class I genes can be confounded by inter- and intralocus recombination. Thus, we combined domain-by-domain phylogenetic analyses with recombination detection methods to examine relationships among MHC class I genes of humans and great apes (Gleimer et al. 2011). Available genomic full-length sequences were aligned using MAFFT and manually edited. Sequence alignments were investigated for patterns of recombination with the program RDP4 which utilizes a variety of methods to identify and detect recombination events (Martin et al. 2015). Based on the results obtained from the RDP4 analysis, MHC class I sequence alignments were divided into various segments, each of which was validated through NJ analyses using the parameters described above.

### Interspecies sequence diversity analyses

To assess variation of MHC class I genes in gorillas, we compared sequence diversity measures of the 33 unrelated gorillas to a similar-sized sample of chimpanzees and humans, respectively. Individual sequence data of 24 chimpanzees were obtained from the study of Adams et al. (2000) which included 19 western chimpanzees (*Pan troglodytes verus*), 3 central chimpanzees (*Pan troglodytes troglodytes*) and two eastern chimpanzees (*Pan troglodytes schweinfurthii*). Genotypes of 30 unrelated Yoruba (YRI) individuals from Ibadan, Nigeria, characterized at the classical *HLA* genes as part of the HapMap project, were taken from the study by de Bakker et al. (2006). Corresponding *HLA* allele sequences were downloaded from the IPD-IMGT/HLA database (Robinson et al. 2015). For each locus separately, pairwise allelic differences of full-length coding region sequences were calculated using MEGA. For the calculation of nucleotide diversity (π), number of variable sites, mean nonsynonymous (Ka) and synonymous (Ks) substitution rates within antigen-binding sites (ABS), we used the program DnaSP version 5.10 (Librado and Rozas 2009). Fisher’s exact test was applied to test the null hypothesis of equal rates of synonymous and nonsynonymous changes.

## Results

**Fig. 1:**
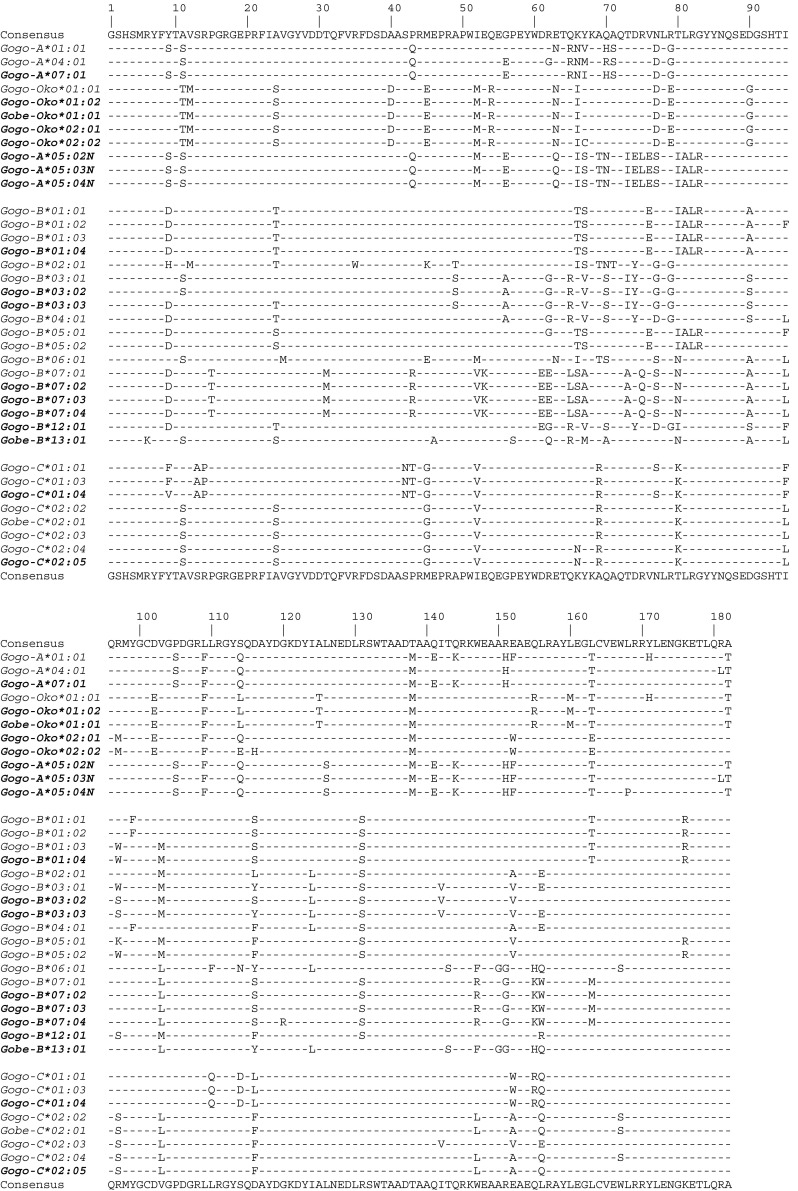
Alignment of amino acid sequences of the alpha-1 and -2 domains of gorilla class I sequences. Identity to consensus sequence is denoted by a *dash*. For differences from the consensus sequence, the amino acid is shown. Highlighted in *bold* are previously unreported alleles. *Gogo*, *Gorilla gorilla*, *Gobe*, *Gorilla beringei*

**Fig. 2:**
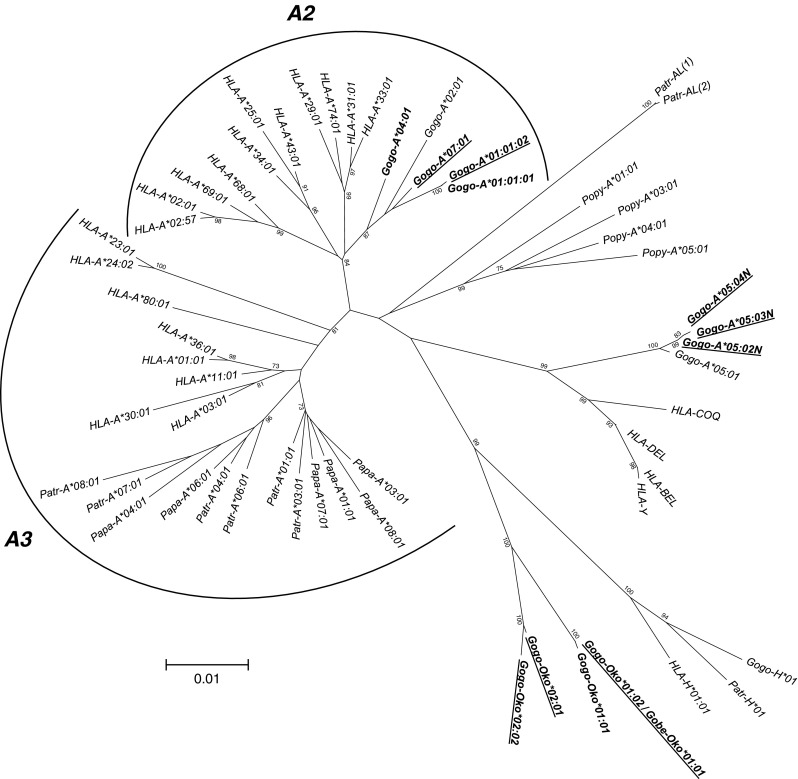
Phylogenetic tree constructed from full-length coding region sequences of *MHC-A* and *-A*-related genes. Relevant bootstrap values (≥70%) are shown. Previously described gorilla alleles identified in the present study are highlighted in *bold*. Newly identified alleles are underlined. (*A2*) and (*A3*) indicate the two phylogenetic *A* lineages, respectively. *HLA* human, *Patr Pan troglodytes*, *Papa Pan paniscus*, *Gogo Gorilla gorilla*, *Popy Pongo pygmaeus*

**Fig. 3:**
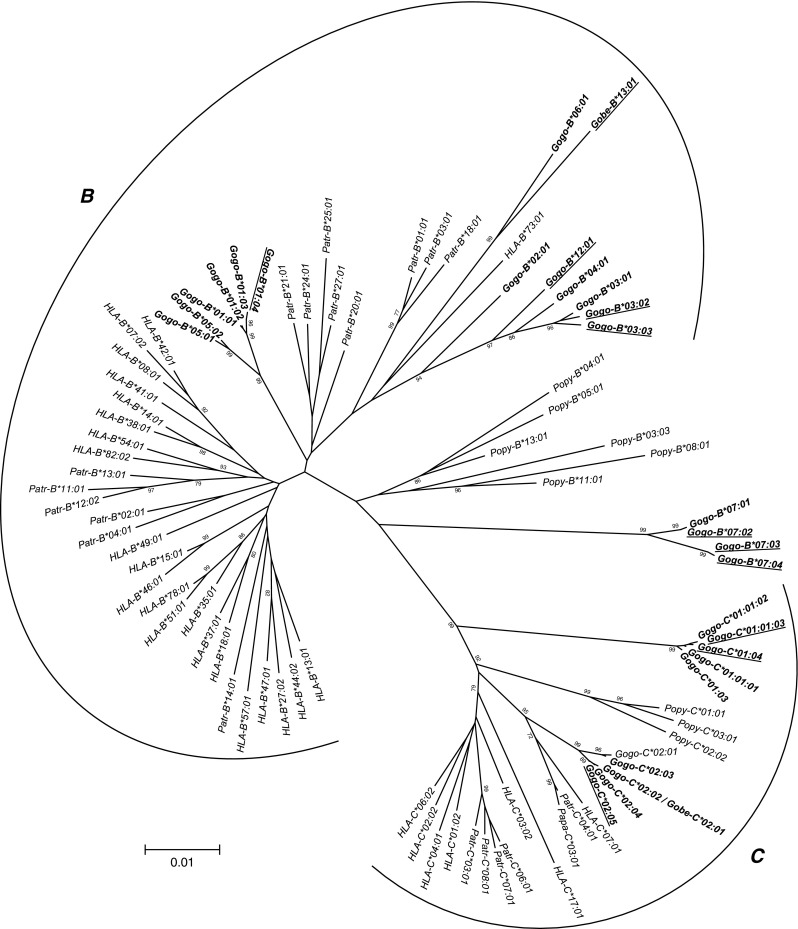
Phylogenetic tree constructed from full-length coding region sequences of *MHC-B* and *-C* genes. Relevant bootstrap values (≥70%) are shown. Previously described gorilla alleles identified in the present study are highlighted in *bold*. Newly identified alleles are underlined. (*B*) and (*C*) indicate the phylogenetic clusters of the orthologous *MHC-B* and *-C* genes, respectively. *HLA* human, *Patr Pan troglodytes*, *Papa Pan paniscus*, *Gogo Gorilla gorilla*, *Gobe Gorilla beringei*, *Popy Pongo pygmaeus*

**Fig. 4:**
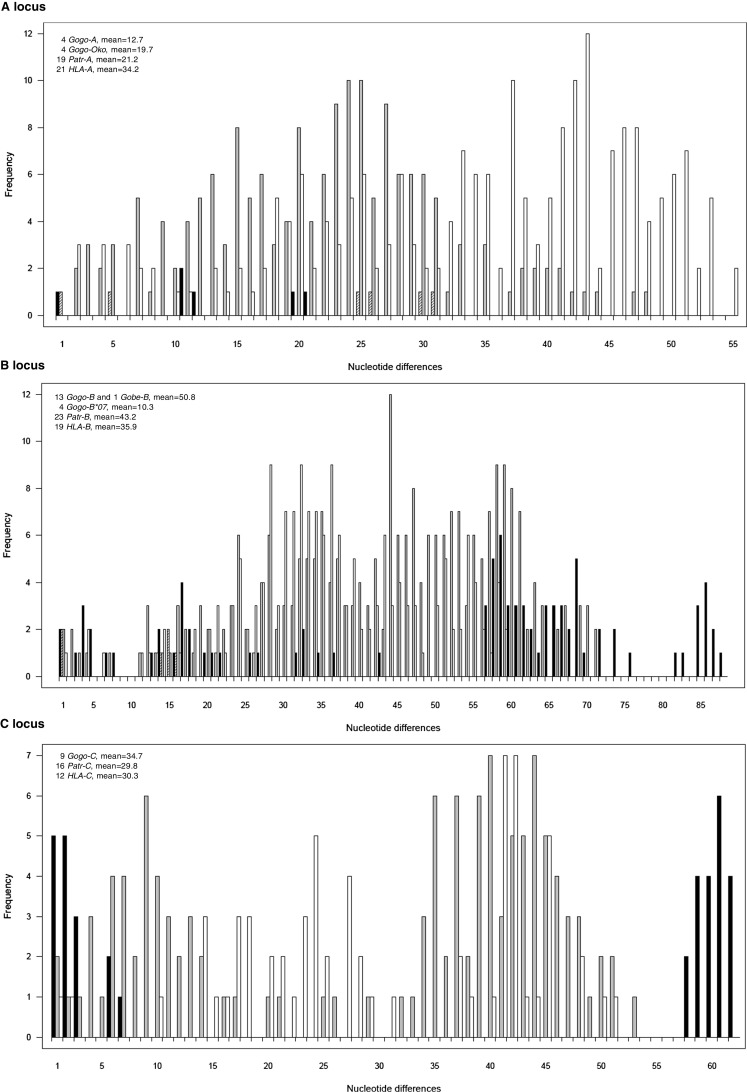
Distribution of pairwise nucleotide differences between complete coding region sequences of MHC class I *A*, *B,* and *C* alleles in gorillas, chimpanzees, and humans. *Gogo*, *Patr*, and *HLA* distributions are shown in *black*, *gray*, and *white*, respectively. *Gogo-Oko* and Gogo-B*07 distributions are shown in *dashed black*, respectively. In the *upper left corner*, the number of alleles used for the calculation of the mean pairwise difference is shown

We used high-quality genomic DNAs from 35 gorillas as templates for individual LR-PCR amplifications of MHC class I genes with primers designed to encompass complete coding region sequences including intervening introns. LR-PCR products were multiplexed and sequenced using PacBio long-read sequencing technology. A total of 50 full-length MHC class I alleles corresponding to 15 *Gogo-A* alleles, 4 *Gogo-Oko*, 21 *Gogo-B* alleles, and 10 *Gogo-C* alleles were identified among the 35 gorillas analyzed. Comparison with previously described *Gogo* class I alleles showed that 19 coding region sequences were novel (Table 1 and Fig. 1). Identification of up to four *Gogo-A* alleles and three *Gogo-B* alleles in certain gorillas indicates the presence of two *Gogo-A* and *-B* genes, respectively (Table 2). Evolutionary relationships of MHC class I genes from gorillas and the other great apes, including humans, were phylogenetically investigated using complete coding region sequences selected to represent the species-specific range of allelic structure at each locus (Figs. 2 and 3). To further examine the evolutionary relationship of the newly identified *Gogo* class I genes, we extended phylogenetic analyses using available full-length genomic MHC class I sequences of human, chimpanzee, and orangutan. Interspecies comparison of sequence diversity measures allowed us to assess the variation at MHC class I genes in gorillas (Fig. 4 and Table 3). Results for each locus will be discussed in turn.

**Table 3:** Interspecies comparison of nucleotide diversity measures. Nucleotide diversity (π) was calculated for complete coding region sequences. Numbers of variable sites were determined across complete coding region sequences and within antigen-binding sites (ABS), respectively. Means of nonsynonymous (Ka) and synonymous (Ks) substitution as well as Ka/Ks ratios (mean of Ka/Ks) were calculated for ABS. Significant differences (*P* < 0.05) between synonymous (Ks) and nonsynonymous (Ka) substitution rates as calculated from Fisher’s exact test are highlighted in italics

Locus	Number of unrelated individuals analyzed	Number of alleles	Nucleotide diversity (π)	Number of variable sites	Ka	Ks	Ka/Ks ratio	*P* value
*Gogo-*	33							
*A*		4	0.01154	22/13	0.05365	0.03747	1.29167	0.5896
*B*		14	0.04667	136/42	0.14401	0.05390	3.03383	*0.0074*
*C*		9	0.03160	66/9	0.03206	0.01328	2.05065	0.2252
*Patr-*	24							
*A*		19	0.01975	67/31	0.07490	0.02858	3.89361	0.1357
*B*		23	0.04054	122/36	0.11703	0.03227	3.71262	*0.0131*
*C*		16	0.02716	86/16	0.04030	0.00800	3.04955	0.0773
*HLA-*	30							
*A*		21	0.04088	70/35	0.09609	0.00355	8.28431	*0.0001*
*B*		19	0.03293	97/31	0.10574	0.01718	5.61417	0.0560
*C*		12	0.02752	97/12	0.03658	0.00527	1.57133	0.3499

### *A* and *A*-related genes in gorillas

Among the 35 gorillas analyzed, we identified 19 full-length alleles corresponding to 15 *Gogo-A* alleles and 4 *Gogo-Oko* alleles (Table 1). Comparison with known *Gogo-A* and *-Oko* alleles showed that 8 of the 11 coding region sequences have not been previously described (Table 1). The novel allele Gogo-A*01:01:02 varied from Gogo-A*01:01 by one synonymous substitution, whereas Gogo-A*07:01 differed at 11 nucleotide positions from Gogo-A*01:01 and -A*04:01, respectively (Lawlor et al. 1991). Interestingly, many gorilla individuals were shown to be heterozygous for Gogo-A*04:01 with differences between alleles observed only in non-coding region sequences. In addition, we identified six individuals being homozygous for either Gogo-A*01:01, -A*04:01, or -A*07:01 alleles (Table 1). Furthermore, three coding region sequences were most similar to the previously described allele Gogo-A*05:01 but differed at 4–6 nucleotide positions (Lawlor et al. 1991). For *Gogo-Oko*, we identified, besides the sole previously described allele, three novel alleles differing at 1–31 nucleotide positions (Lawlor et al. 1991; Watkins et al. 1991). Among the 35 gorillas analyzed, we identified three individuals homozygous for *Gogo-Oko* alleles (Table 2). Some *Gogo-A* and *-Oko* alleles are shared between gorilla species and subspecies as expected given the trans-species polymorphism of MHC genes (reviewed in Klein et al. 1998) (Table 1). In this context, it has to be noted that the allele Gobe-Oko*01:01 identified in the eastern lowland gorilla is identical to the Gogo-Oko*01:02 allele found in western lowland gorillas. Overall, translation of the 11 coding region sequences resulted in 10 unique amino acid sequences. However, comparison of the three polypeptide chains obtained from the four alleles Gogo-A*01:01:01, -A*01:01:02, -A*04:01, and -A*07:01 revealed very similar sequences suggesting low functional variation (Fig. 1). In contrast to the previously described Gogo-A*05:01 allele, we identified in each of the three novel Gogo-A*05 alleles a nonsynonymous substitution resulting in a stop codon at position 257 within the exon 4, presumably causing a loss of function. Indeed, identical nonsense mutations were identified in the null alleles HLA-A*02:15N, -A*24:183N, and -A*02:356N (Ishikawa et al. 1996; Shimizu et al. 2013). These findings strongly suggest that molecules of Gogo-A*05 alleles from the present study are nonfunctional and therefore, were denoted by the suffix N. Similar to the *HLA* null alleles, it is unlikely that identified Gogo-A*05 alleles are expressed due to their lack of the transmembrane and intracellular regions encoded by the exons downstream of the stop codon. That in a previous study, cDNA was obtained for a Gogo-A*05 null allele in the gorilla “Beta” but not in the gorilla “Banga” argues for varying levels of messenger RNA (mRNA) transcription which, however, does not contradict our conclusions (Lawlor et al. 1991; Urvater et al. 2001) (Table 2). For *Gogo-Oko*, however, translation of the four alleles resulted in four unique amino acid sequences indicating allelic polymorphism.

In the phylogenetic tree (Fig. 2), coding region sequences group alleles of *Gogo-A* (gorilla), *Patr-A* (chimpanzee), *Papa-A* (bonobo), and *HLA-A* (human) together indicating that this clade represents the *A* orthologue of gorilla, chimpanzee, bonobo, and human. Within this clade, alleles are divided into two groups, the ancient lineages *A2* and *A3* (Lawlor et al. 1990). Although *HLA-A* alleles segregate in both lineages, the two novel and three previously described alleles Gogo-A*01:01, -A*01:01:02, -A*02:01, -A*04:01, and -A*07:01 segregate only into the *A2* lineage together with alleles of the *HLA-A19* family. In contrast, *Patr-A* and *Papa-A* alleles group only into the *A3* lineage with alleles of the *HLA-A1*/*A3*/*A11* family, as has been shown (Lawlor et al. 1995; McAdam et al. 1995; Adams et al. 2000; de Groot et al. 2000). The previously described Gogo-A*05:01 allele and the novel Gogo-A*05 alleles, however, fall outside of both the *A2* and *A3* lineages instead forming a cluster with the human pseudogene *HLA-Y*, previously known as *HLA-BEL*, *-COQ*, and -*DEL* (Williams et al. 1999; Coquillard et al. 2004; Marsh et al. 2010). Contrary to previous suggestions, Gogo-A*05 alleles cannot represent a divergent *A* lineage because we identified individual gorillas which have Gogo-A*05 alleles in addition to two *Gogo-A* alleles showing that these are definitely separate genes (Table 2) (Lawlor et al. 1991; Coquillard et al. 2004). Thus, our findings strongly suggest that Gogo-A*05 represents the gorilla orthologue of *HLA-Y*. Similarly, alleles of the *Gogo-Oko* locus are phylogenetically distinct, segregating with *H* locus alleles of human, chimpanzee, and gorilla consistent with previous findings showing that *Gogo-Oko*, through recombination, shares partial sequence homology with the pseudogene *H* (Lawlor et al. 1991; Watkins et al. 1991; Adams and Parham 2001; Gleimer et al. 2011). Thus, our results indicate that gorillas possess, besides *Gogo-A* and *Gogo-Oko*, an additional *A*-related locus consisting of Gogo-A*05 alleles which likely represents a pseudogene in the gorillas analyzed.

To further examine the evolutionary relationship of the novel Gogo-A*05 with *Gogo-A*, *-Oko*, and related MHC class I genes of the other hominid species, we performed domain-by-domain phylogenetic analyses using available genomic full-length sequences (Online Resource 4). As expected, Gogo-A*05 is most closely related to the human pseudogene *HLA-Y*. Except for exon 3 and its flanking introns which show higher sequence similarity to other *Gogo-A* locus alleles, Gogo-A*05 is orthologous to *HLA-Y* throughout the gene. Thus, our results clearly show that Gogo-A*05 is the gorilla orthologue of *HLA-Y*. In this context, *HLA-Y* has been suggested to be the human equivalent of the chimpanzee *A*-related locus *Patr-AL* (Gleimer et al. 2011). Consistent with previous findings, domain-by-domain phylogenetic analyses demonstrate that both *HLA-Y* and Gogo-A*05 have segments orthologous to *Patr-AL* (Gleimer et al. 2011). In the 5′ part which includes the 5′ flanking region, exon 1, intron 1, and partial exon 2, both *HLA-Y* and Gogo-A*05 have sequences in common with *Patr-AL*. Otherwise, *HLA-Y* and Gogo-A*05 share sequence similarity with *A* locus alleles of the *A2* lineage. In contrast, *Patr-AL* is most closely related to the orangutan *Popy-A* which likewise shares sequence similarity with *HLA-Y* and Gogo-A*05 in the 5′ flanking region. Furthermore, *Gogo-Oko* appears to be orthologous to *Popy-A*/Gogo-A*05/*Patr-AL*/*HLA-Y* in the 5′ flanking region, and indeed, analysis of a gorilla MHC haplotype revealed that *Gogo-Oko* is located in the same genomic position as *Patr-AL* (Gleimer et al. 2011). Otherwise, *Gogo-Oko* shares sequence similarity with the nonfunctional *H* and other *A* locus alleles consistent with previous findings (Lawlor et al. 1991; Watkins et al. 1991; Adams and Parham 2001; Gleimer et al. 2011). Taken together, our results, in combination with previous findings, demonstrate the orthologous relationship of the *A*-related genes in human (*HLA-Y*), chimpanzee (*Patr-AL*), gorilla (Gogo-A*05 and *Gogo-Oko*), and orangutan (*Popy-A*).

To assess the gorilla MHC class I diversity, we compared allelic and sequence variability to a similar-sized sample of chimpanzees and humans. Based on the number of *MHC-A* alleles, gorillas are much less diverse than chimpanzees and humans. Among the 33 unrelated gorillas analyzed, we identified only 4 different coding region sequences for *Gogo-A* whereas a similar sized study of chimpanzees (*n* = 24) identified 19 different *Patr-A* alleles. Among 30 unrelated Yoruba individuals, a total of 21 *HLA-A* alleles have been described. Further evidence showing that the *MHC-A* diversity in gorillas is reduced compared to chimpanzees and humans, comes from the distribution of pairwise allelic differences (Fig. 4). The mean pairwise difference of *Gogo-A* alleles (mean = 12.7) is much lower than the means of *Patr-A* alleles (mean = 21.2) and *HLA-A* alleles (mean = 34.2), respectively. The reduced diversity in gorillas is not only influenced by the absence of *A3* lineage alleles but is also due to the homogeneity of *Gogo-A* alleles, as implied by Figs. 1 and 4. In contrast, chimpanzees appear to have accumulated variation at *Patr-A* despite their lack of alleles belonging to the *A2* lineage. The distribution of pairwise differences between *HLA-A* alleles, however, reflects the presence of *A2* and *A3* lineage alleles in the Yoruba population. As expected from the comparison of pairwise allelic differences, nucleotide diversity was lowest for *Gogo-A* (*π* = 0.01154) compared to *Patr-A* (*π* = 0.01975) and *HLA-A* (*π* = 0.04088), respectively (Table 3). The overall numbers of variable sites of coding region sequences were 22, 67, and 70 for gorillas, chimpanzees and humans, respectively. Within the antigen-binding sites (ABS), numbers of variable sites were 13 for *Gogo-A* alleles, 31 for *Patr-A* alleles, and 35 for *HLA-A* alleles (Table 3). Although Fisher’s exact test showed no significant differences between substitution rates at *Gogo-A*, the nonsynonymous substitution rate (Ka) is substantially lower in gorillas compared to chimpanzees and humans. The synonymous substitution rate (Ks), however, appears to be highest in gorillas which might suggest that selection acts against amino acid altering mutations at *Gogo-A* (Table 3). In contrast, the Ka/Ks ratio in humans is strikingly higher compared to gorillas and chimpanzees which can be attributed to the significantly lower synonymous substitution rate at *HLA-A* (Table 3).

### B genes in gorillas

For *Gogo-B*, we identified a total of 21 genomic full-length alleles corresponding to 18 different coding region sequences (Table 1). Comparison with previously described *Gogo-B* alleles revealed that 8 of the 18 coding region sequences were novel. The allele Gogo-B*01:04 differed from Gogo-B*01:03 by one nonsynonymous substitution, whereas coding region sequences of Gogo-B*03:02 and -B*03:03 differed from Gogo-B*03:01 at five and seven nucleotide positions, respectively (Lawlor et al. 1991; Urvater et al. 2001). The novel allele Gogo-B*07:02 varied from Gogo-B*07:01 by one nonsynonymous substitution (Abi-Rached et al. 2011). On the contrary, the alleles Gogo-B*07:03 and -B*07:04 were most similar to Gogo-B*07:01 but shared sequence similarity in exons 5–7 with other *Gogo-B* alleles, a likely result of interallelic gene conversion. The novel allele Gogo-B*12:01 was most similar to Gogo-B*04:01 differing at 20 nucleotide positions, whereas the allele Gobe-B*13:01, which was only observed in the eastern lowland gorillas, differed from the previously described allele Gogo-B*06:01 at 35 nucleotide positions (Abi-Rached et al. 2011). Unlike *Gogo-A* and *-Oko* alleles, we identified only one *Gogo-B* allele shared between the two western gorilla subspecies, suggesting the rapid diversification of *B* locus alleles (Table 1). The 18 different coding region sequences translated into 18 unique amino acid sequences. Comparison of polypeptide chains revealed that many amino acid motifs are shared between alleles indicating that recombination plays an important role in the generation of allelic variation (Fig. 1).

The phylogenetic tree including full-length *MHC-B* coding region sequences shows little substructure with alleles predominantly forming species-specific clusters (Fig. 3). The absence of clearly defined *B* lineages has been attributed to frequent recombination and interlocus gene conversion at this MHC locus, as reflected by low bootstrap support values (Lawlor et al. 1990; Adams and Parham 2001). Consistent with previous findings, alleles of human, chimpanzee, gorilla, and orangutan mostly group by species with the exception of certain gorilla and chimpanzee *B* alleles segregating with HLA-B*73:01 to form a clade which has been suggested to represent an ancient *B* lineage (Parham et al. 1994; Hoffmann et al. 1995; Abi-Rached et al. 2011; Yasukochi and Ohashi 2016). Distinguishing the ancient *B* lineage, all these alleles share a 3-bp insertion in exon 5 which adds another amino acid to the transmembrane region (Adams and Parham 2001). Included in this clade are the gorilla *B* alleles Gogo-B*02:01, -B*03:01, -B*03:02, -B*03:03, -B*04:01, -B*06:01, -B*12:01, and Gobe-B*13:01 whereas the alleles Gogo-B*01:01, -B*01:02, -B*01:03, -B*01:04, -B*05:01, and -B*05:02 form a separate cluster within the clade that consists of the other human and chimpanzee *B* alleles. Despite the relatively few *MHC-B* alleles found in gorillas, our findings indicate that gorillas have two distinct *B* loci because we identified seven individuals each of which possessing three *Gogo-B* alleles (Table 2). Divergent from the other *Gogo-B* alleles, the alleles Gogo-B*07:01, -B*07:02, -B*07:03, and -B*07:04 clearly segregate apart and appear to be most closely related to *Popy-B* alleles of orangutans. Strong linkage disequilibrium between alleles of Gogo-B*03 and -B*07 demonstrates that these are located on the same haplotype and in accordance, we identified one individual which was homozygous for Gogo-B*03 and -B*07, respectively (Table 2). Our data thus suggest that, in addition to *Gogo-B*, gorillas possess another *B* locus which is present only on some gorilla *B* haplotypes.

To further examine the evolutionary relationships of the novel *Gogo-B* gene, we extended our phylogenetic analyses by using available genomic full-length DNA sequences of human, chimpanzee, and orangutan (Online Resource 5). Domain-by-domain analyses of full-length sequences show that, as expected, alleles of Gogo-B*03 co-segregating with Gogo-B*07 are most similar to other gorilla *B* alleles. In the 5′ region, Gogo-B*03 alleles clearly segregate with Gogo-B*04:01 whereas in the remaining part of the gene Gogo-B*03 alleles form a cluster with Gogo-B*02:01, -B*04:01 and -B*12:01 indicating that Gogo-B*03 alleles belong to the gorilla *B* orthologue of *HLA-B* and *Patr-B*. The novel Gogo-B*07 locus, however, has a complex recombinant structure. In the 5′ region and the intron 3, Gogo-B*07 alleles appear to be most similar to the orangutan *B* locus allele Popy-B*03:02 whereas there is sequence similarity to *MHC-C* alleles in the region encompassing exon 2 and 3 and its intervening intron. In exon 4 and intron 4, however, Gogo-B*07 alleles are related to other gorilla *B* locus alleles. The phylogenetic tree constructed from exon 5 to 8 sequences segregates Gogo-B*07:01 and -B*07:02 alleles together with Popy-B*03:02 distinct from Gogo-B*07:03 and -B*07:04 alleles which are most closely related to alleles of the ancient *B* lineage. Accordingly, the Gogo-B*07:03 and -B*07:04 alleles share the 3-bp insertion in exon 5 characteristic for the ancient *B* lineage suggesting that Gogo-B*07:03 and -B*07:04 acquired the 3′ region through interallelic gene conversion with *Gogo-B* alleles of the ancient *B* lineage. Thus, there is evidence to suggest that alleles of Gogo-B*07 represent the orthologue of *Popy-B* which can be found in multiple copies in individual orangutans (Chen et al. 1992; Adams et al. 1999; de Groot et al. 2016).

As described in detail above, some gorillas possess an additional *Gogo-B* gene which has no orthologue in the *MHC* of chimpanzee and human, respectively. Thus, for a comparable assessment of the *MHC-B* diversity in gorillas, chimpanzees, and humans, Gogo-B*07 alleles were excluded from sequence diversity analyses. Among the 33 unrelated gorillas, we identified a total of 14 different coding region sequences at *Gogo-B*. In contrast, a study of 24 chimpanzees revealed 23 *Patr-B* alleles (Table 3). Allelic variation at *HLA-B* is represented by 19 alleles which were characterized among 30 unrelated Yoruba individuals. Thus, based on the number of alleles, gorillas have lower variation at the orthologous *MHC-B* gene compared to chimpanzees and humans. As seen from the histograms in Fig. 4, the distribution of pairwise allelic differences is considerably different in gorillas as compared to chimpanzees and humans. This is predominantly due to the prevalence of divergent *Gogo-B* alleles, as reflected by phylogenetic patterns in Fig. 3. In contrast, distributions of pairwise allelic differences at *Patr-B* and *HLA-B* indicate that allelic diversity accumulated within *MHC-B* lineages of chimpanzees and humans, respectively. Consistent with the divergence of *Gogo-B* alleles, nucleotide diversity at *MHC-B* is higher in gorillas (*π* = 0.04667) than in chimpanzees (*π* = 0.04054) and humans (*π* = 0.03293) (Table 3). The numbers of variable sites within full-length coding region sequences of *MHC-B* alleles are 136, 122, and 97 for gorillas, chimpanzees, and humans, respectively. Within the ABS region, numbers of variable sites are 42, 36, and 31 for *Gogo-B*, *Patr-B*, and *HLA-B*, respectively. Calculation of mean Ka and Ks within the ABS shows significantly higher substitution rates in gorillas than in chimpanzees (Table 3). However, the higher Ka/Ks ratios at *Patr-B* is due to the significantly lower synonymous substitution rate suggesting that positive selection in chimpanzees is acting more effectively in altering amino acid residues than in gorillas. In contrast, Fisher’s exact test revealed no significant differences between synonymous (Ks) and nonsynonymous substitution rates at *HLA-B* (Table 3).

### C gene in gorillas

Among the 35 gorillas analyzed, we identified a total of 10 *Gogo-C* full-length genomic sequences corresponding to nine different coding region sequences (Table 1). Comparison with known *Gogo-C* alleles showed that three coding region sequences were not described previously. The novel allele Gogo-C*01:01:03, which was only observed in the Cross River gorilla, differed from Gogo-C*01:01 by two synonymous substitutions (Lawlor et al. 1991). The novel allele Gogo-C*01:04 varied from Gogo-C*01:01 by one nonsynonymous and one synonymous substitution. Please also note that the previously described allele Gogo-C*01:02 was renamed to Gogo-C*01:01:02 because it varies from Gogo-C*01:01:01 at one synonymous nucleotide position (Lawlor et al. 1991). Differing by one nonsynonymous substitution, the novel allele Gogo-C*02:05 was most closely related to Gogo-C*02:04 which, however, has not been described in full-length (Urvater et al. 2001). As with the *A* and *A*-related loci, trans-species polymorphism of *Gogo-C* alleles was observed among the gorilla species and subspecies, all of which shared the allele Gogo-C*02:02 (Table 1). However, this allele has been designated as Gobe-C*02:01 in the eastern lowland gorilla. The nine different coding region sequences of *Gogo-C* alleles translated into seven unique amino acid sequences. Comparison of amino acid sequences revealed the existence of two *Gogo-C* lineages with intra-allelic variation mainly generated through point mutations (Fig. 1).

Within the phylogenetic tree in Fig. 3, coding region sequences of the *MHC-C* gene in human, chimpanzee, bonobo, gorilla, and orangutan clearly segregate within a distinct clade indicating their orthologous relationship. Besides species-specific clustering of alleles, phylogenetic patterns suggest the presence of conserved *C* lineages across species. As such, the gorilla alleles Gogo-C*02:01, -C*02:02, -C*02:03, -C*02:04, and -C*02:05 as well as certain chimpanzee and bonobo *C* alleles cluster together with HLA-C*07:01 (Zemmour and Parham 1992; Adams and Parham 2001). On the contrary, the gorilla alleles Gogo-C*01:01:01, -C*01:01:02, -C*01:01:03, -C*01:03, and -C*01:04 form a gorilla-specific branch within the *C* locus clade. However, the majority of *Patr-C* and *HLA-C* alleles segregate together into one allelic lineage which appears to be absent from gorillas.

Our study of 33 unrelated gorillas revealed nine different coding region sequences at the *Gogo-C* locus (Table 3). The study of 24 chimpanzees by Adams et al. (2000) characterized 16 different *Patr-C* alleles. Among the 30 unrelated individuals of the Yoruba population, a total of 12 *HLA-C* alleles were identified. Thus, gorillas appear to have lower diversity at the *C* locus with regard to number of alleles. The histogram of pairwise allelic differences at *Gogo-C* is substantially different from that of chimpanzees and humans (Fig. 4). As expected, the distribution of pairwise differences between *Gogo-C* alleles reflects the presence of only two divergent lineages with intra-allelic variation predominantly generated through point mutations. In contrast, the histograms of chimpanzees and humans reveal the presence of multiple lineages, as can be seen from the distribution of pairwise differences between *Patr-C* and *HLA-C* alleles. In accordance, nucleotide diversity is higher at *Gogo-C* (*π* = 0.03160) than at *Patr-C* (*π* = 0.02716) and *HLA-C* (*π* = 0.02752). However, the number of variable sites, both within coding regions and the ABS, is substantially lower in gorillas (66/9) compared to chimpanzees (86/16) and humans (97/12). Although Fisher’s exact test showed no significant differences between synonymous and nonsynonymous substitution rates in humans, chimpanzees and gorillas, respectively, it appears that gorillas have maintained diversity at functionally important ABS as suggested by an intermediate Ka/Ks ratio (Table 3).

### Gogo-B and -C allotypes for KIR recognition

**Fig. 5:**
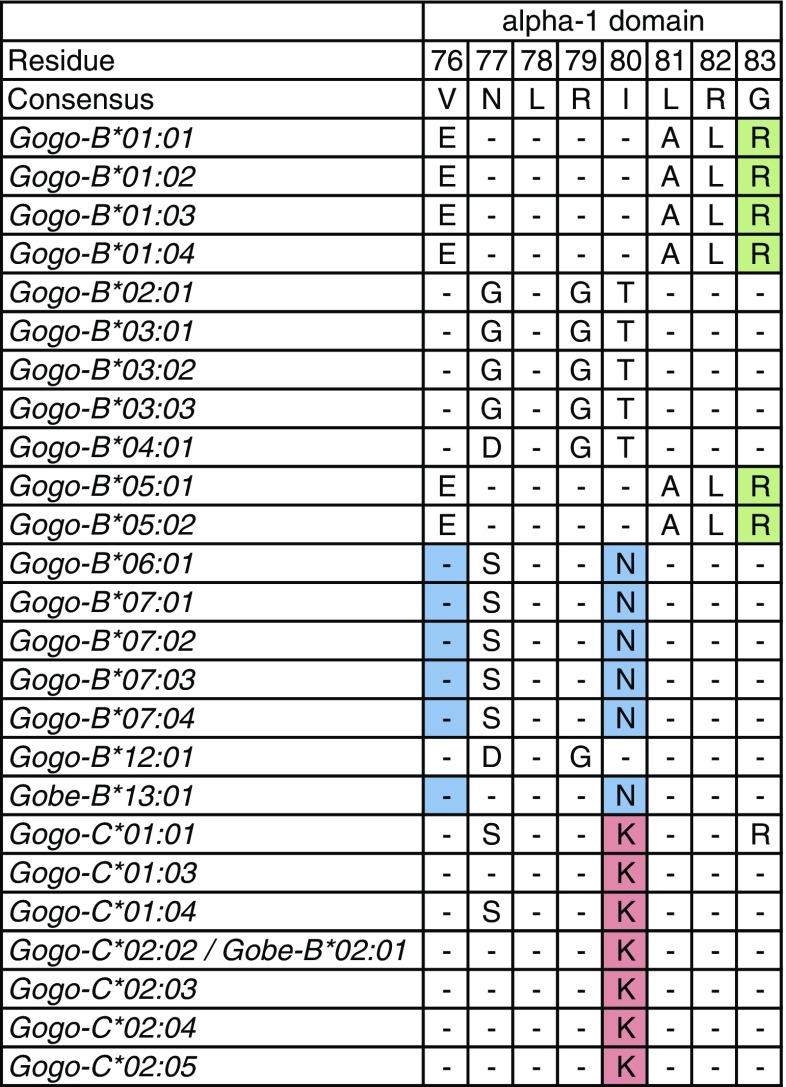
Amino acid sequences of *Gogo-B* and *-C* alleles determining the KIR-binding epitope. Identity to consensus sequence is denoted by a *dash*. For differences from the consensus sequence, the amino acid is shown. Positions highlighted in *green*, *blue*, and *red* define the Bw4 epitope, the C1 and the C2 epitopes, respectively

KIR recognition of MHC class I epitopes is determined by amino acid residues 76–83 in the alpha-1 domain. Lineage II KIR receptors interact with the Bw4 epitope expressed by MHC-B allotypes which is defined by arginine (R) at position 83 while expression of valine (V) at position 76 and asparagine (N) at position 80 defines the C1 epitope which is recognized by lineage III KIR receptors. MHC-B allotypes with other sequence motifs at positions 76–83 are not ligands for KIR receptors (Parham and Moffett 2013). In our study, 12 of the 18 Gogo-B allotypes are predicted to be ligands for KIR receptors. All of the Gogo-B*01 and -B*05 allotypes have the Bw4 epitope while Gogo-B*06:01 and Gobe-B*13:01 as well as all allotypes of Gogo-B*07 have the C1 epitope. The remaining six Gogo-B allotypes have neither the Bw4 epitope nor the C1 epitope (Fig. 5). In contrast, recognized by lineage III KIR receptors, MHC-C allotypes are distinguished by a dimorphism at amino acid position 80. The C1 epitope is carried by MHC-C allotypes that have asparagine (N) at position 80 while the C2 epitope is carried by MHC-C allotypes with lysine (K) at position 80 (Parham and Moffett 2013). Interestingly, all Gogo-C allotypes identified are predicted to have the motif which defines the C2 epitope (Fig. 5).

## Discussion

In the present study, we studied 35 gorillas yielding 15 *Gogo-A* alleles, 4 *Gogo-Oko*, 21 *Gogo-B* alleles, and 10 *Gogo-C* alleles. We identified two previously undetected genes related to *Gogo-A* and *Gogo-B* in some of the individuals. Data obtained in gorillas were analyzed in the context of other available information from humans, chimpanzees, bonobos, and orangutans. Our results provide evidence for gorillas having a comparatively complex MHC class I haplotype structure but the overall MHC class I variation appears to be low.

The previous, albeit small, sampling of *Gogo* class I alleles showed that *Gogo-A* alleles segregate only into the *A2* lineage suggesting that *A3* lineage alleles are lacking in gorillas (Lawlor et al. 1991; Watkins et al. 1991; Cadavid and Watkins 1997; Adams and Parham 2001). Indeed, among the 35 gorillas analyzed, we identified only *Gogo-A* alleles of the *A2* lineage (Fig. 2). Whereas in humans, the *A2* lineage has diversified considerably and now includes the three divergent families *HLA-A2*, *-A10*, and *-A19*; gorillas have only equivalents of the *HLA-A19* family (Fig. 2) (Lawlor et al. 1990). Equally diverse in humans is the *A3* lineage with the three families *HLA-A1/A3/A11*, *-A9*, and *-A80* whereas the *A* locus alleles of chimpanzees and bonobos, *Patr-A* and *Papa-A*, segregate only into the *HLA-A1*/*A3*/*A11* family (Parham et al. 1989; Lawlor et al. 1990, 1995; Domena et al. 1993; McAdam et al. 1995; Adams et al. 2000; de Groot et al. 2000). This reduction of the chimpanzee and bonobo MHC class I repertoire has been attributed to a pathogen-mediated selective sweep which caused the extinction of *A2* lineage alleles in the ancestral *Pan* population (de Groot et al. 2002, 2008). However, whether the *A3* lineage emerged after the split of humans and chimpanzees from gorillas or was present in their common ancestor and subsequently lost during gorilla evolution remains to be elucidated. In this context, previous studies revealed that gorillas show comparatively little variability at MHC class II genes as well as a marked reduction of genetic diversity in regions flanking the MHC indicative of a recent selective sweep (Scally et al. 2013; Hans et al. 2015; Xue et al. 2015). Thus, our findings further support the hypothesis that gorillas might have experienced a reduction of their MHC diversity. Indeed, the small number of high-frequency alleles associated with an increased homozygosity suggests that the reduction at *Gogo-A* has occurred quite recently (Tables 1 and 2). In accordance, gorillas appear to have accumulated only limited variation both within coding and non-coding region sequences, as reflected by an interspecies comparison of allelic variation (Table 3 and Fig. 4). Thus, our findings strongly suggest that gorillas recently underwent an episode of increased selective pressure which might have resulted in the low *Gogo-A* diversity.

Interestingly, some gorillas possess an additional and putatively functional *A*-related locus, designated *Gogo-Oko*, which has been shown to be located in the same genomic position as *Patr-AL* (Lawlor et al. 1991; Watkins et al. 1991; Gleimer et al. 2011). However, distinguishing *Gogo-Oko* is the unusual structure of its corresponding gorilla MHC haplotype. Whereas *Patr-AL* co-segregates with *Patr-A*, the gorilla MHC haplotype with *Gogo-Oko* is characterized by the absence of the genomic block containing the *A* gene (Adams et al. 2001; Geller et al. 2002; Gleimer et al. 2011). In accordance, we identified three individuals homozygous for *Gogo-Oko* (Table 2). Besides the sole previously described *Gogo-Oko* allele, we characterized three novel alleles suggesting that *Gogo-Oko* evolved to become a classical MHC class I gene. Indeed, evidence that *Gogo-Oko* gene products are involved in the antigen presentation comes from the study by Watkins et al. (1991), which demonstrated its expression in 7 of 13 gorillas (53.8%). We observed a similar distribution with 16 of 35 gorillas (45.7%) possessing *Gogo-Oko* suggestive of balancing selection maintaining this haplotype at intermediate frequencies in the population. We thus propose that *Gogo-Oko*, at least in part, compensates for the low diversity at the *Gogo-A* locus.

Besides *Gogo-Oko*, we here show that gorillas possess another *A*-related gene which is represented by Gogo-A*05 alleles. Despite previous suggestions, alleles of Gogo-A*05 cannot represent a divergent *Gogo-A* lineage because we identified individual gorillas which have Gogo-A*05 alleles in addition to two *Gogo-A* alleles showing that these are definitely separate genes (Table 2) (Lawlor et al. 1991; Coquillard et al. 2004). Domain-by-domain phylogenetic analyses clearly demonstrate that Gogo-A*05 is the equivalent of the human pseudogene *HLA-Y* and our findings suggest that Gogo-A*05 likewise represents a pseudogene in the gorillas analyzed (Online Resource 4) (Williams et al. 1999; Coquillard et al. 2004; Marsh et al. 2010). A nonsense mutation in exon 4 argues that the Gogo-A*05 alleles identified are nonfunctional. Similarly, the *HLA-Y* alleles, *-COQ*, and *-DEL*, are nonfunctional due to nonsense mutations in exon 4 whereas *HLA-Y*, also designated as *HLA-BEL*, lacks the exon 3 in addition to a dual-cytosine insertion in exon 4 causing a frameshift and premature termination (Williams et al. 1999; Coquillard et al. 2004). Due to their structural and functional similarities, we propose to rename its gorilla equivalent as *Gogo-Y*.

**Fig. 6:**
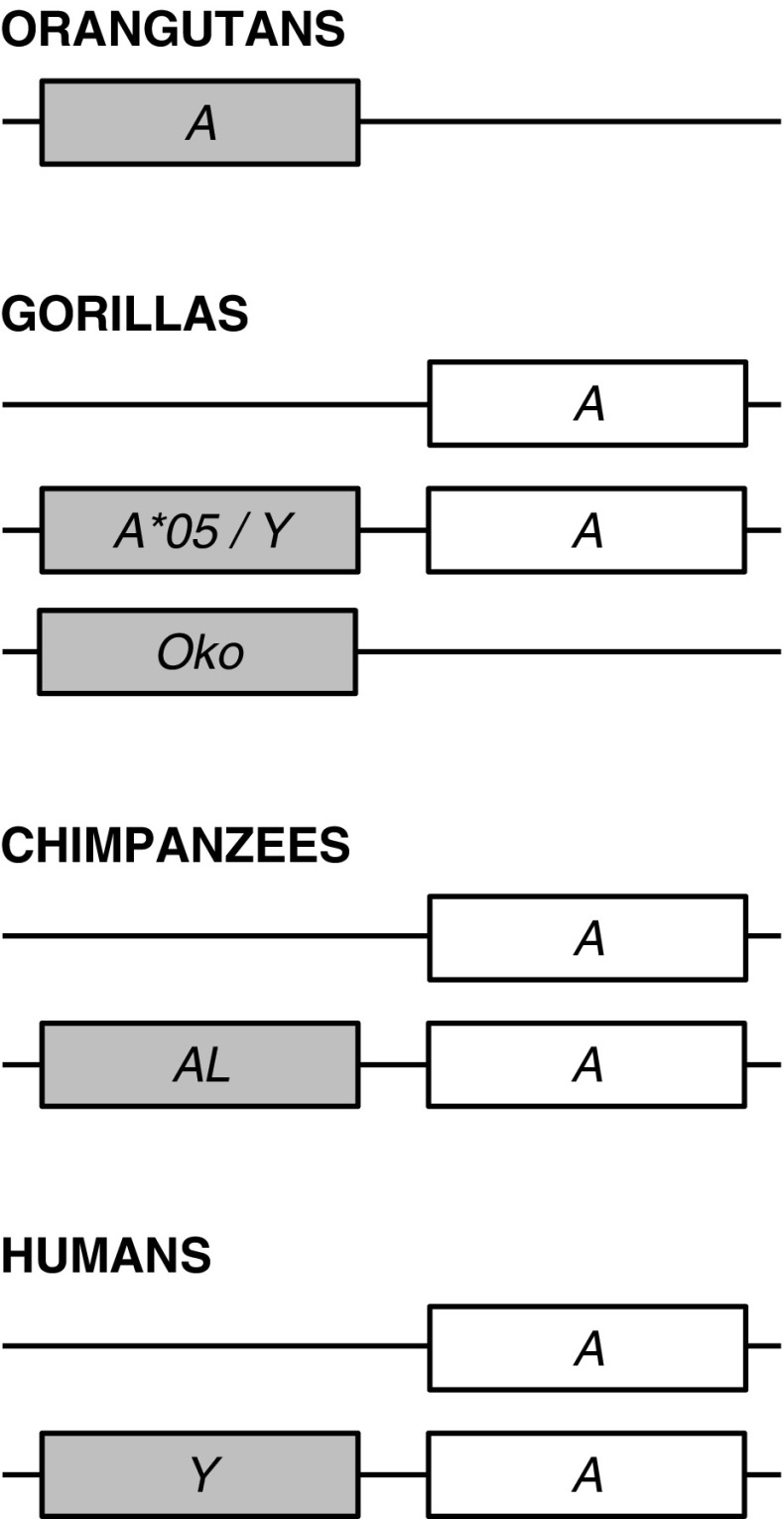
Simplified MHC class I *A* region haplotypes of humans and the great apes. *White boxes* represent the orthologous *MHC-A* gene whereas *gray boxes* represent the orthologous *MHC-A*-related gene

Our analyses further show that *Gogo-Y* is orthologous to the 5′ part of the functional *A*-related genes *Patr-AL* in chimpanzees and *Popy-A* in orangutans indicating that these genes shared a common ancestor before the hominid diversification (Online Resource 4) (Adams et al. 2001; Gleimer et al. 2011; Goyos et al. 2015). That the orthologous *A*-related (pseudo)genes in human and gorilla independently acquired nonsense mutations argues for selection that has led to their inactivation, possibly associated with disease susceptibility. However, *Gogo-Y* is also orthologous to the 5′ flanking region of *Gogo-Oko* suggesting that the ancestral *A*-related gene in gorillas evolved differently to become nonfunctional and classical (Online Resource 4). As is the case for *Gogo-Oko*, our findings suggest that *Gogo-Y* is not present on all gorilla MHC haplotypes although linkage disequilibrium to *Gogo-A* demonstrates its location within the MHC class I region, as determined through patterns of allelic inheritance among related individuals (Table 2). Similarly, *HLA-Y* and *Patr-AL* are present only on a subset of the human and chimpanzee MHC haplotypes despite their co-segregation with the orthologous *A* locus (Fig. 6) (Williams et al. 1999; Adams et al. 2001; Geller et al. 2002; Coquillard et al. 2004; Gleimer et al. 2011). Interestingly, the presence of *HLA-Y* is predominantly associated with alleles of the *HLA-A19* family and it is this family to which the orthologous *Gogo-A* alleles are most closely related (Williams et al. 1999; Coquillard et al. 2004; Gleimer et al. 2011). Thus, it appears that this MHC haplotype has remained stable since humans and gorillas last shared a common ancestor. In nucleotide sequence, both *HLA-Y* and *Gogo-Y* share sequence similarity with *A2* lineage alleles whereas *Gogo-Oko* through recombination obtained segments from *H* and other primate *A* locus alleles (Online Resource 4) (Lawlor et al. 1991; Watkins et al. 1991; Adams and Parham 2001; Gleimer et al. 2011). In contrast, *Patr-AL* is most closely related to *Popy-A*, and for that reason it has been suggested that true orthologues of the *A* locus are present only in humans (*HLA-A*) and the African great apes, chimpanzees (*Patr-A*), bonobos (*Papa-A*), and gorillas (*Gogo-A*), but not orangutans (Fig. 6) (Adams et al. 2001; Gleimer et al. 2011). Consistent with our findings in gorillas, an evolutionary model has been proposed in which the *A* and *A*-related genes descended from a common ancestor by two successive duplications followed by partial deletions forming the extant hominid MHC haplotypes (Gleimer et al. 2011). As such, the deletion of the genomic block with the *A*-related gene produced the *A* haplotype which now can be found in humans, chimpanzees, bonobos and gorillas (Fig. 6) (Watanabe et al. 1997; Gleimer et al. 2011). On the contrary, deletion of the genomic block with the *A* locus formed the *A*-related haplotype represented only by *Gogo-Oko* and absent from humans and the other hominid species (Fig. 6) (Gleimer et al. 2011). Thus, our results in combination with previous findings show that gorillas have a comparatively complex MHC class I region although the overall functional diversity appears to be low at the *A* and *A*-related genes.

In contrast, the previous characterization of 18 alleles of *Gogo-B*, the gorilla orthologue of *HLA-B*, suggested a high degree of polymorphism at this MHC class I locus (Lawlor et al. 1991; Urvater et al. 2001; Martínez-Laso et al. 2006; Abi-Rached et al. 2011). Our study of 35 gorillas identified a total of 17 full-length *Gogo-B* alleles, five of which had novel coding region sequences (Table 1). Phylogenetically, *Gogo-B* alleles group into two distinct clusters whereas the *HLA-B* and *Patr-B* alleles show a dispersed distribution with multiple species-specific branches (Fig. 3). These patterns are also evident from the distributions of pairwise allelic differences reflecting the prevalence of divergent alleles in gorillas as compared to chimpanzees and humans (Fig. 4). The majority of *Gogo-B* alleles segregate with certain *Patr-B* alleles and the HLA-B*73:01 allele forming an ancestral *B* lineage shared between humans, chimpanzees and gorillas while the remaining *Gogo-B* alleles group phylogenetically into a gorilla-specific branch of the clade (Parham et al. 1994; Abi-Rached et al. 2011; Yasukochi and Ohashi 2016). Included in the latter are alleles of Gogo-B*01 which were identified in 20 of the 35 gorillas analyzed (57.1%). In this context, Gogo-B*01 alleles have been suggested to be responsible for the high prevalence of spondyloarthropathy (SpA) in gorillas, an inflammatory disorder primarily affecting the spine (Rothschild and Woods 1989; Urvater et al. 2001). Indeed, although divergent in their nucleotide sequences, Gogo-B*01 molecules have peptide-binding repertoires similar to HLA-B*27 which is a strong risk factor for SpA in humans (Urvater et al. 2001). Molecules of HLA-B*27 and -B*57 have also been shown to give a protective effect in HIV-1 infection (Kiepiela et al. 2004; Fellay et al. 2007). In particular, HLA-B*27 and -B*57 alleles expressing the Bw4 epitope and isoleucine (I) at amino acid residue 80 in combination with the activating KIR II lineage allele KIR3DS1 are associated with a delayed disease progression to AIDS (Martin et al. 2002). Interestingly, like the protective *HLA-B* allotypes, Gogo-B*01 and -B*05 alleles are predicted to express the Bw4 epitope and isoleucine at position 80 (Fig. 5). That two groups of HIV-1 originated through cross-species transmission of SIV from gorillas (SIVgor) to humans highlights the possibility of gorillas having MHC class I molecules which provide resistance to lentiviral infections (Van Heuverswyn et al. 2006; Plantier et al. 2009; D’arc et al. 2015). However, it is unknown whether SIVgor infections are pathogenic in its gorilla host (Sharp and Hahn 2011; but see Moeller et al. 2015). The other two groups of HIV-1, including its fatal form which develops into AIDS in humans, descended from SIV in chimpanzees (SIVcpz) (Keele et al. 2006). However, in contrast to humans, chimpanzees naturally infected with SIVcpz are less likely to develop AIDS-like symptoms (reviewed in Sharp and Hahn 2011; but see Pandrea et al. 2009; Keele et al. 2009). This relative resistance of chimpanzees has been attributed to a selective sweep which was caused by an AIDS-like pandemic in the ancestral *Pan* population (de Groot et al. 2002). As a consequence, AIDS-protective MHC class I alleles were selected and increased in frequency in modern chimpanzees, a process that was, however, accompanied by a general reduction of the *Patr* class I repertoire (de Groot et al. 2002, 2008; de Groot and Bontrop 2013). Indeed, it has been shown that the most frequent *Patr* class I molecules recognize similar HIV/SIVcpz epitopes as the AIDS-protective HLA-B*27/B*57 in humans (de Groot et al. 2010; see also Wroblewski et al. 2015). Thus, there is evidence to suggest that in gorillas, the comparatively little MHC class I diversity likewise resulted from a pathogen-mediated selective sweep.

Despite the apparent low *Gogo-B* diversity, we show that certain gorillas possess an additional *B* locus because we identified seven individuals which have three *Gogo-B* alleles (Table 2). Divergent from the other gorilla *B* locus alleles, Gogo-B*07 alleles fall outside of the orthologous *B* clade shared between humans, chimpanzees, bonobos, and gorillas and appear to be most closely related to *Popy-B* which exhibits diverse copy number variation in orangutans (Fig. 3) (Chen et al. 1992; Adams et al. 1999; de Groot et al. 2016). Indeed, domain-by-domain phylogenetic analyses show that the newly identified Gogo-B*07 gene shares partial sequence similarity with *Popy-B* suggesting that Gogo-B*07 represents an ancestral *B* lineage predating the hominid divergence (Online Resource 5). In contrast to most other *Gogo-B* alleles which either have the Bw4 epitope or do not interact with KIR receptors, alleles of Gogo-B*07 are predicted to carry the C1 epitope (Fig. 5). Thus, it appears that gorillas have maintained the putative ancestral *B* locus on certain MHC haplotypes due to its important function in controlling NK cell activity via KIR receptors.

**Fig. 7:**
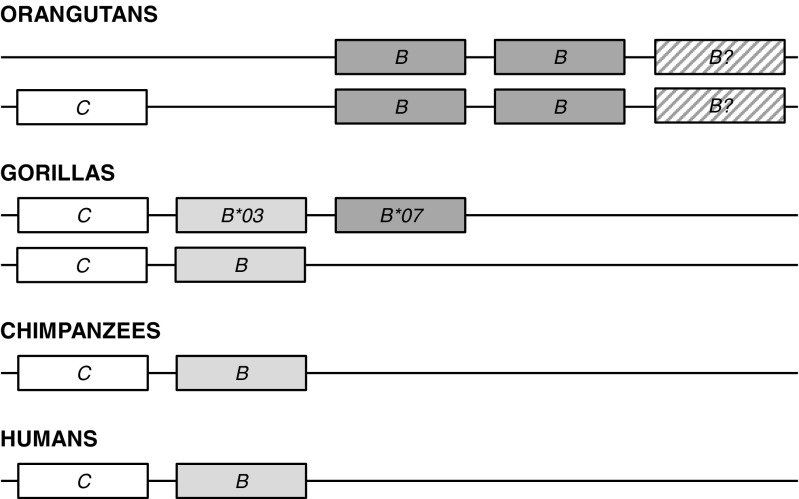
Simplified MHC class I *B* and *C* region haplotypes of humans and the great apes. *White boxes* represent the orthologous *MHC-C* gene whereas *light gray boxes* represent the orthologous *MHC-B* gene. *Dark gray boxes* represent the orthologous *MHC-B* gene present only in gorillas and orangutans. Copy number variation at the *MHC-B* gene in orangutans is depicted by a *question mark*

However, the dominant ligand for KIR receptors in humans is MHC-C and its comparatively low polymorphism has been attributed to the coevolution with KIR receptors (Adams and Parham 2001; Vilches and Parham 2002; Single et al. 2007; Older Aguilar et al. 2010). In humans and chimpanzees, all MHC haplotypes have the *MHC-C* gene whereas in orangutans, it is present only on some of the MHC haplotypes (Adams et al. 1999; de Groot et al. 2016). Similar to its fixation in humans and chimpanzees, our findings suggest that *Gogo-C*, the gorilla orthologue of *HLA-C*, is present on all gorilla MHC haplotypes (Table 2). Among the 35 gorillas, we identified a total of 10 full-length *Gogo-C* alleles including three coding region sequences that have not been previously described (Table 1). Phylogenetically, *Gogo-C* alleles clearly segregate with *C* locus alleles of human, chimpanzee, and bonobo as well as orangutan indicating their orthologous relationship (Fig. 3). However, *Gogo-C* alleles group phylogenetically only into two lineages with polymorphism mainly generated through point mutations as evidenced by the distribution of pairwise allelic differences, thus suggesting low functional variation (Figs. 3 and 4). Indeed, Gogo-C allotypes are predicted to express only one of the two epitopes that are recognized by lineage III KIR receptors, the C2 epitope, whereas in humans and chimpanzees MHC-C allotypes with both the C1 and C2 epitopes have been identified (Fig. 5) (Adams and Parham 2001; Moesta et al. 2009). In contrast, orangutan MHC-C allotypes carry only the C1 epitope indicating that it was present in the common ancestor of humans and the great apes (Guethlein et al. 2002, 2007). The C2 epitope, however, emerged with the fixation of the *MHC-C* gene, and in accordance, is carried by MHC-C allotypes of gorillas, chimpanzees, and humans but not orangutans (Parham and Moffett 2013; Guethlein et al. 2015). Thus, there is evidence to suggest that gorillas have lost the C1-bearing allotypes of MHC-C. The C1 epitope is also carried by some orangutan, chimpanzee, and human MHC-B allotypes indicating that it was also present in the ancestral *MHC-B-like* gene that duplicated to produce two paralogous MHC class I genes, now recognized as *MHC-B* and *MHC-C* (Fukami-Kobayashi et al. 2005). In gorillas, the C1 epitope is predominantly carried by the newly identified Gogo-B*07 gene. Interestingly, exons 2 and 3 and the intervening intron of Gogo-B*07 are most closely related to *MHC-C* which suggests that Gogo-B*07 represents the remnant of the *MHC-B-like* ancestor that was maintained by selection on certain gorilla and orangutan MHC haplotypes but lost in the common ancestor of *Pan* and humans (Fig. 7). Taken together, there is evidence for a complex history of the hominid *MHC-B* and *MHC-C* genes which cannot be explained by an evolutionary model involving a single duplication event.

In sum, our study demonstrates the utility of characterizing a larger sample of gorillas for a more complete understanding of the MHC class I gene variability. We identified that gorillas have a comparatively complex MHC class I haplotype structure with characteristics shared between humans and chimpanzees as well as orangutans, thereby providing new insights into the evolution of the MHC class I genes in humans and the great apes. However, the apparently low diversity suggests that gorillas might have experienced a reduction of their MHC class I repertoire, presumably through a selective sweep, which should be investigated in more detail in future studies.

## Electronic supplementary material

ESM 1 (PDF 31 kb)

ESM 2 (PDF 82 kb)

ESM 3 (PDF 51 kb)

ESM 4 (PDF 682 kb)

ESM 5 (PDF 605 kb)
