# Supplementary material for: Gorilla MHC class I gene and sequence variation in a comparative context
Source: Immunogenetics. 2017 Mar 22;69(5):303–23. doi: 10.1007/s00251-017-0974-x (PMC5400801; doi:10.1007/s00251-017-0974-x)
Supplement: Supplementary file 1 — (PDF 31 kb) [file 251_2017_974_MOESM1_ESM.pdf]

Electronic Supplementary Material 1. Sample information

Species	*Common name*	*Name*	*Studbook ID*	*Sex*	*Geographical origin/birthplace*	*Birth origin*	*Reference*
Gorilla beringei graueri	Eastern lowland gorilla	Mukisi	9912	M	Walikale region, Democratic Republic of the Congo	Wild born	Jensen-Seaman and Kidd (2001)
Gorilla gorilla diehli	Cross River gorilla	Nyango	9941	F	Mamfe (Cross River region), Cameroon	Wild born	Thalmann et al. (2007); Good et al. (2013)
Gorilla gorilla gorilla	Western lowland gorilla	Makulla	0051	F	Guinea (Equatorial?)	Wild born	Good et al. (2013); Wilms and Bender (2015)
Gorilla gorilla gorilla	Western lowland gorilla	Willie B	0115	M	Cameroon	Wild born	Wilms and Bender (2015)
Gorilla gorilla gorilla	Western lowland gorilla	Samson II	0137	M	Congo	Wild born	Wilms and Bender (2015)
Gorilla gorilla gorilla	Western lowland gorilla	Beta	0160	F	Unknown	Wild born	Wilms and Bender (2015)
Gorilla gorilla gorilla	Western lowland gorilla	Ozoum	0175	M	Unknown	Wild born	Wilms and Bender (2015)
Gorilla gorilla gorilla	Western lowland gorilla	Paki	0191	F	Unknown	Wild born	Wilms and Bender (2015)
Gorilla gorilla gorilla	Western lowland gorilla	Oko	0192	F	Unknown	Wild born	Wilms and Bender (2015)
Gorilla gorilla gorilla	Western lowland gorilla	Banga	0224	F	Unknown	Wild born	Wilms and Bender (2015)
Gorilla gorilla gorilla	Western lowland gorilla	Donna	0336	F	Cameroon	Wild born	Wilms and Bender (2015)
Gorilla gorilla gorilla	Western lowland gorilla	Carlos	0506	M	Unknown	Wild born	Wilms and Bender (2015)
Gorilla gorilla gorilla	Western lowland gorilla	Machi	0609	F	Yerkes National Primate Center, GA, USA	Captive born	Wilms and Bender (2015)
Gorilla gorilla gorilla	Western lowland gorilla	Murphy	0684	M	Henry Doorly Zoo and Aquarium, NE, USA	Captive born	Wilms and Bender (2015)
Gorilla gorilla gorilla	Western lowland gorilla	Rok	0701	M	Yerkes National Primate Center, GA, USA	Captive born	Wilms and Bender (2015)
Gorilla gorilla gorilla	Western lowland gorilla	Ivan	0710	M	Republic of Congo	Wild born	Wilms and Bender (2015)
Gorilla gorilla gorilla	Western lowland gorilla	Gorgo	0766	M	Zoo Krefeld, Germany	Captive born	Wilms and Bender (2015)
Gorilla gorilla gorilla	Western lowland gorilla	N'Diki	0858	F	Cameroon	Wild born	Wilms and Bender (2015)
Gorilla gorilla gorilla	Western lowland gorilla	Bebe France	0860	F	Cameroon	Wild born	Good et al. (2013); Wilms and Bender (2015)
Gorilla gorilla gorilla	Western lowland gorilla	Binti Jua	1047	F	Columbus Zoo and Aquarium, OH, USA	Captive born	Wilms and Bender (2015)
Gorilla gorilla gorilla	Western lowland gorilla	Kwan	1107	M	North Carolina Zoo, NC, USA	Captive born	Wilms and Bender (2015)
Gorilla gorilla gorilla	Western lowland gorilla	Bulera	1120	F	Lincoln Park Zoo, IL, USA	Captive born	Wilms and Bender (2015)
Gorilla gorilla gorilla	Western lowland gorilla	Baraka Y Mwelu	1273	M	Smithsonian National Zoological Park, DC, USA	Captive born	Wilms and Bender (2015)
Gorilla gorilla gorilla	Western lowland gorilla	Abeeku	1516	M	Diergaarde Blijdorp, NL	Captive born	Wilms and Bender (2015)
Gorilla gorilla gorilla	Western lowland gorilla	Chella aka Rambo	1912	M	Cameroon	Wild born	Thalmann et al. (2007); Wilms and Bender (2015)
Gorilla gorilla gorilla	Western lowland gorilla	Emma	1914	F	East Province, Cameroon	Wild born	Thalmann et al. (2007); Good et al. (2013); Wilms and Bender (2015)
Gorilla gorilla gorilla	Western lowland gorilla	Jumbo	1916	F	Bertoua, East Province, Cameroon	Wild born	Thalmann et al. (2007); Good et al. (2013); Wilms and Bender (2015)
Gorilla gorilla gorilla	Western lowland gorilla	Pitchou	1925	F	Lolodore-Lolodorf, South West, Cameroon	Wild born	Thalmann et al. (2007); Wilms and Bender (2015)
Gorilla gorilla gorilla	Western lowland gorilla	Akiba	1926	F	East Province (120 km from Belabo), Cameroon	Wild born	Thalmann et al. (2007); Good et al. (2013); Wilms and Bender (2015)
Gorilla gorilla gorilla	Western lowland gorilla	Batek	1927	M	Batouri, East Province, Cameroon	Wild born	Thalmann et al. (2007); Good et al. (2013); Wilms and Bender (2015)
Gorilla gorilla gorilla	Western lowland gorilla	Brighter	1928	F	Cameroon	Wild born	Wilms and Bender (2015)
Gorilla gorilla gorilla	Western lowland gorilla	Twigs	1929	F	Cameroon	Wild born	Wilms and Bender (2015)
Gorilla gorilla gorilla	Western lowland gorilla	Anthal	1930	F	South Province (village: Nkoma, subdivision: Mvangan, division: Muilla), Cameroon	Wild born	Thalmann et al. (2007); Good et al. (2013); Wilms and Bender (2015)
Gorilla gorilla gorilla	Western lowland gorilla	Arnaud	1931	M	South Province (village: Nkoma, subdivision: Mvangan, division: Muilla), Cameroon	Wild born	Thalmann et al. (2007); Good et al. (2013); Wilms and Bender (2015)
Gorilla gorilla gorilla	Western lowland gorilla	EB (JC)	Cat. 89072703	F	Unknown	Unknown	

References

Good JM, Wiebe V, Albert FW, Burbano HA, Kircher M, Green RE, Halbwax M, André C, Atencia R, Fischer A, Pääbo S (2013) Comparative population genomics of the ejaculate in humans and the great apes. Mol Biol Evol 30:964-976. doi: 10.1093/molbev/mst005

Jensen-Seaman MI, Kidd KK (2001) Mitochondrial DNA variation and biogeography of eastern gorillas. Mol Ecol 10:2241-2247

Thalmann O, Fischer A, Lankester F, Pääbo S, Vigilant L (2007) The complex evolutionary history of gorillas: insights from genomic data. Mol Biol Evol 24:146-158

Wilms TM, Bender U (2015) International studbook for the western lowland gorilla Gorilla g. gorilla Savage & Wyman, 1847. Frankfurt Zoo, Frankfurt
