# Supplementary material for: Gorilla MHC class I gene and sequence variation in a comparative context
Source: Immunogenetics. 2017 Mar 22;69(5):303–23. doi: 10.1007/s00251-017-0974-x (PMC5400801; doi:10.1007/s00251-017-0974-x)
Supplement: Supplementary file 2 — (PDF 82 kb) [file 251_2017_974_MOESM2_ESM.pdf]

Electronic Supplementary Material 2. Genealogies of captive born gorillas

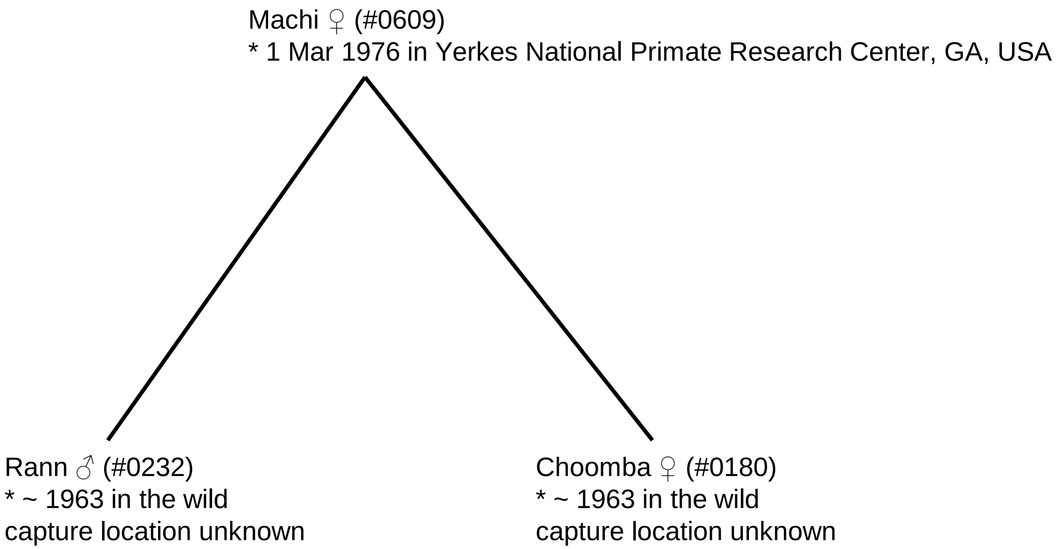

Murphy ♂ (#0684)
* 17 Dec 1978 in Henry Doorly Zoo and Aquarium, NE, USA

Casey I ♂ (#0083)
* ~ 1956 in the wild
captured in West Africa

Bridgette ♀ (#0226)
* ~ 1961 in the wild
captured in West Africa

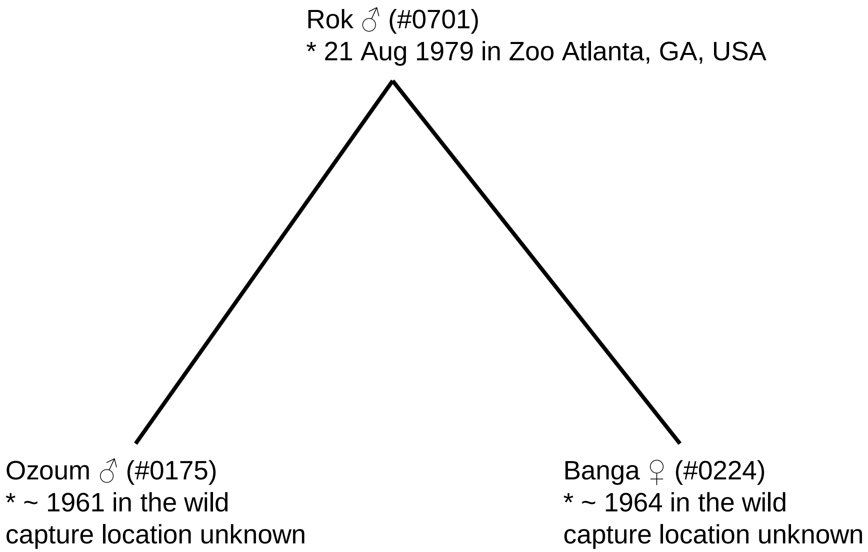
Rok ♂ (#0701)
* 21 Aug 1979 in Zoo Atlanta, GA, USA
Ozoum ♂ (#0175)
* ~ 1961 in the wild
capture location unknown
Banga ♀ (#0224)
* ~ 1964 in the wild
capture location unknown

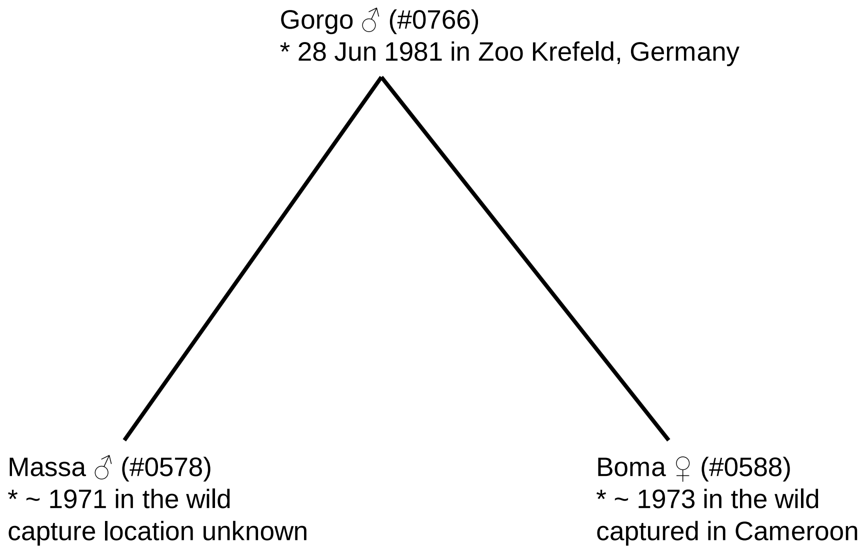
Gorgo ♂ (#0766)
* 28 Jun 1981 in Zoo Krefeld, Germany
Massa ♂ (#0578)
* ~ 1971 in the wild
capture location unknown
Boma ♀ (#0588)
* ~ 1973 in the wild
captured in Cameroon

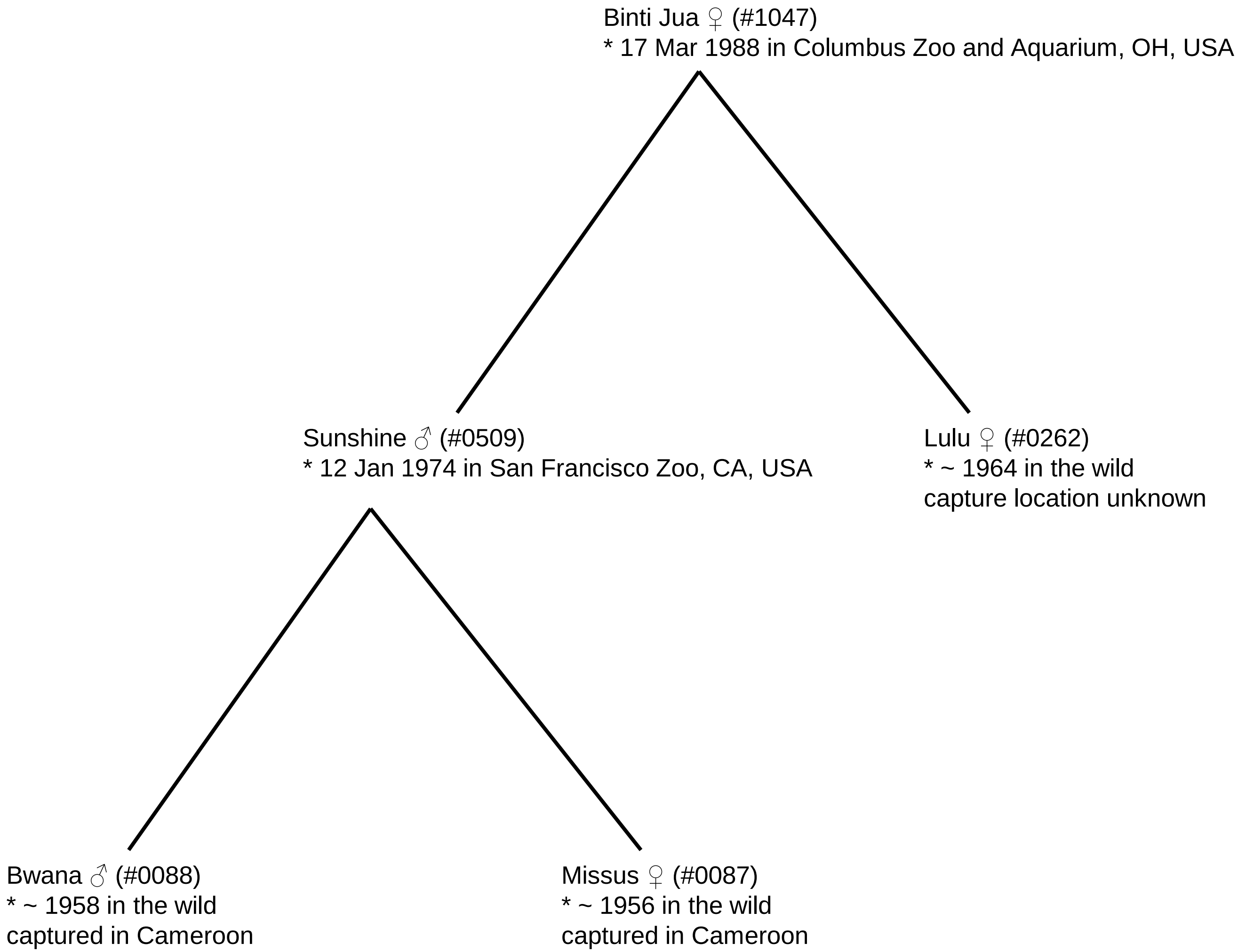
Binti Jua ♀ (#1047)
* 17 Mar 1988 in Columbus Zoo and Aquarium, OH, USA
Sunshine ♂ (#0509)
* 12 Jan 1974 in San Francisco Zoo, CA, USA
Lulu ♀ (#0262)
* ~ 1964 in the wild
capture location unknown
Bwana ♂ (#0088)
* ~ 1958 in the wild
captured in Cameroon
Missus ♀ (#0087)
* ~ 1956 in the wild
captured in Cameroon

Kwan ♂ (#1107)
* 02 Mar 1989 in North Carolina Zoo, NC, USA

Carlos ♂ (#0506)
* ~ 1970 in the wild
capture location unknown

Hope ♀ (#0559)
* 30 Oct 1974 in Gulf South Research Institute, FL, USA

Jimmie Gee ♂ (#0388)
* ? in the wild
capture location unknown

Sheila ♀ (#0390)
* ? in the wild
capture location unknown

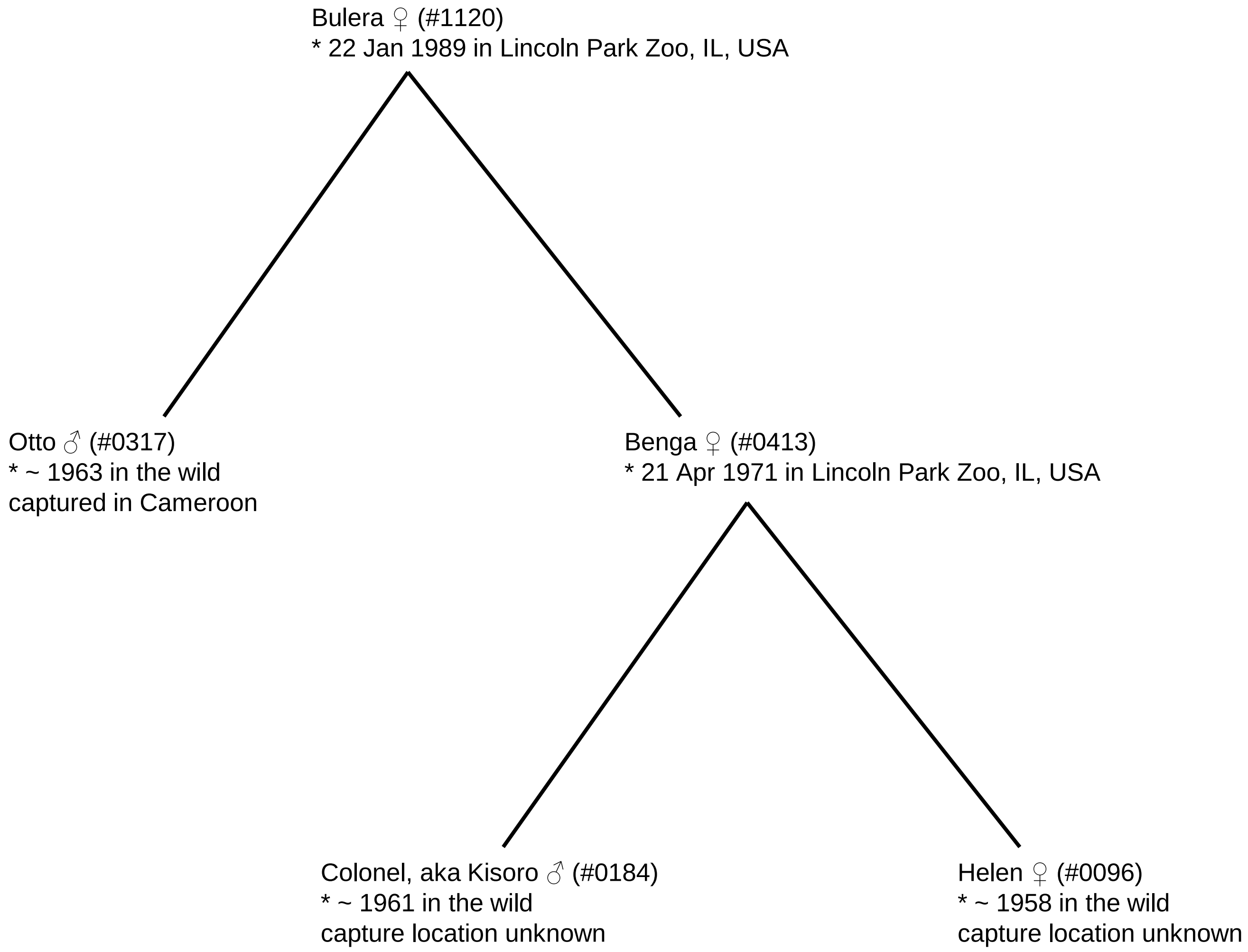
Bulera ♀ (#1120)
* 22 Jan 1989 in Lincoln Park Zoo, IL, USA
Otto ♂ (#0317)
* ~ 1963 in the wild
captured in Cameroon
Benga ♀ (#0413)
* 21 Apr 1971 in Lincoln Park Zoo, IL, USA
Colonel, aka Kisoro ♂ (#0184)
* ~ 1961 in the wild
capture location unknown
Helen ♀ (#0096)
* ~ 1958 in the wild
capture location unknown

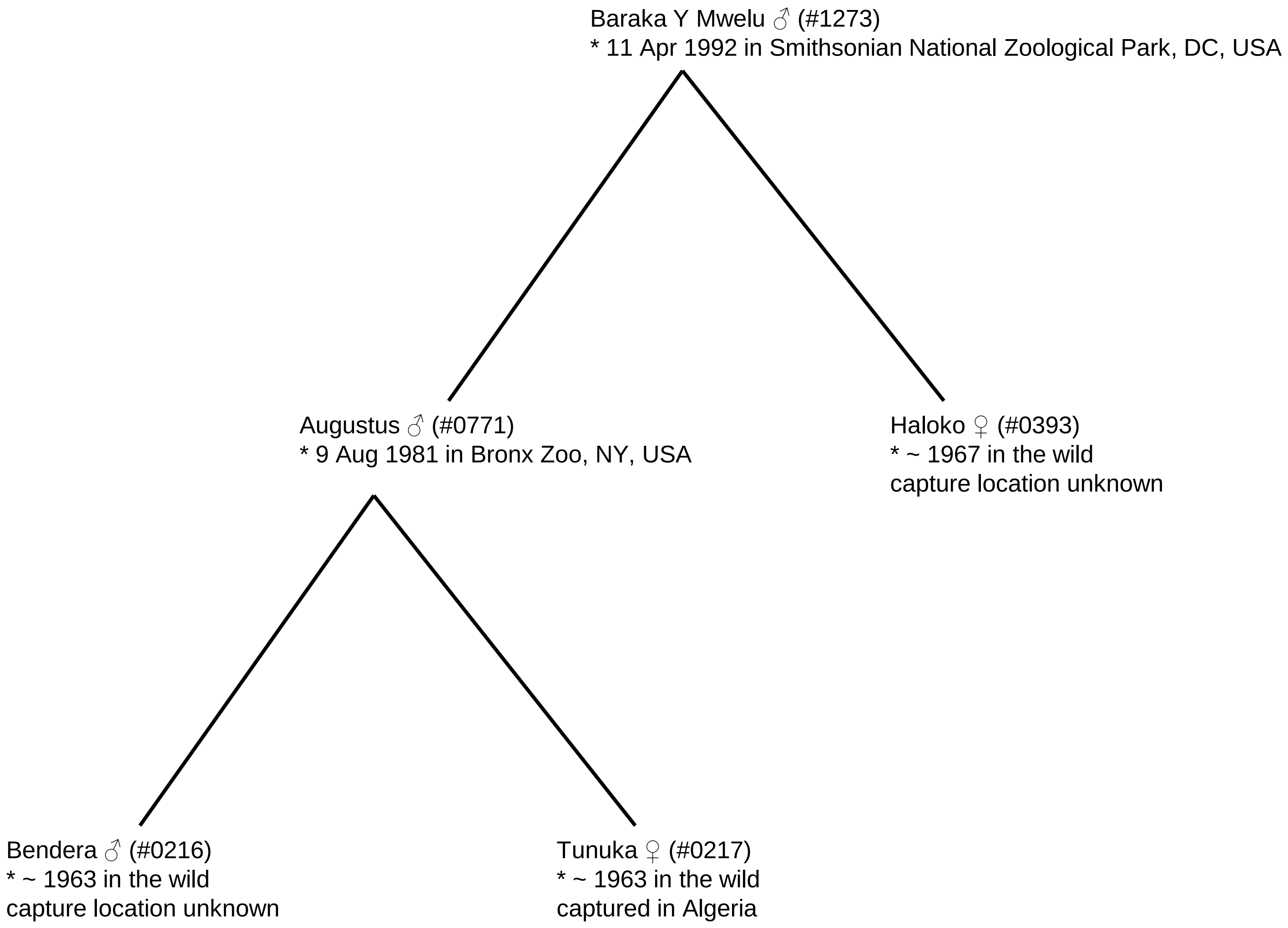
Baraka Y Mwelu ♂ (#1273)
* 11 Apr 1992 in Smithsonian National Zoological Park, DC, USA
Augustus ♂ (#0771)
* 9 Aug 1981 in Bronx Zoo, NY, USA
Haloko ♀ (#0393)
* ~ 1967 in the wild
capture location unknown
Bendera ♂ (#0216)
* ~ 1963 in the wild
capture location unknown
Tunuka ♀ (#0217)
* ~ 1963 in the wild
captured in Algeria

Abeeku ♂ (#1516)
* 5 May 1999 in Diergaarde Blijdorp, NL

Ernst ♂ (#0566)
* ~ 1972 in the wild
captured in Cameroon

Annette ♀ (#0586)
* ~ 1973 in the wild
captured in Cameroon

Reference
Wilms TM, Bender U (2015) International studbook for the western lowland gorilla Gorilla g. gorilla Savage & Wyman, 1847. Frankfurt Zoo, Frankfurt
