# Supplementary material for: Gorilla MHC class I gene and sequence variation in a comparative context
Source: Immunogenetics. 2017 Mar 22;69(5):303–23. doi: 10.1007/s00251-017-0974-x (PMC5400801; doi:10.1007/s00251-017-0974-x)
Supplement: Supplementary file 3 — (PDF 51 kb) [file 251_2017_974_MOESM3_ESM.pdf]

Electronic Supplementary Material 3. Primer pairs used for the amplification of gorilla MHC class I genes

Locus	***Primer name***	***Sequence (5'-3')***	***Expected PCR product size in bp***
Gorilla *MHC-A/Oko*	Gogo_A/Oko_F	GCGGACMCAGTTCTCACTSCCATTG	~ 3160/3207
	Gogo_A/Oko_R	GCCTACARGAACACAGACACATKCAGG	
Gorilla *MHC-B*	Gogo_B_F	GTCGKGTCCTTCTTCCRGGATACTCG	3020-3033
	Gogo_B_R	GRCAGCTGTCTCAGGCTACAGAAAACAAC	
Gorilla *MHC-C*	Gogo_C_F	CGWCGGGTCCTTCTTCCTGAATACTCA	3079
	Gogo_C_R	TGCATCTCAGTCCCACACAGGCAG	
