# Supplementary material for: Gorilla MHC class I gene and sequence variation in a comparative context
Source: Immunogenetics. 2017 Mar 22;69(5):303–23. doi: 10.1007/s00251-017-0974-x (PMC5400801; doi:10.1007/s00251-017-0974-x)

Electronic Supplementary Material 4. Domain-by-domain phylogenetic analysis of the newly identified *Gogo-A*05* gene. Neighbor-joining trees were constructed from available full-length genomic sequences of *MHC-A* and -*A*-related genes. Shown in the top left corner is the size of the genomic segments (1-6) which were used for the phylogenetic reconstruction. Black, grey and white circles correspond to bootstrap values of ≥95%, ≥80% or ≥50%, respectively. Black triangles represent compressed *MHC-A* sequences of human (*H*), chimpanzee (*C*) and gorilla (*G*) with the total number of sequences given in parentheses. Highlighted in red are sequences of *Gogo-A*05* alleles. Sequences of other gorilla alleles identified in the present study are highlighted in bold. *HLA*, human; *Patr*, *Pan troglodytes*; *Gogo*, *Gorilla gorilla*; *Gobe*, *Gorilla beringei*; *Popy*, *Pongo pygmaeus*; *Mamu*, *Macaca mulatta*

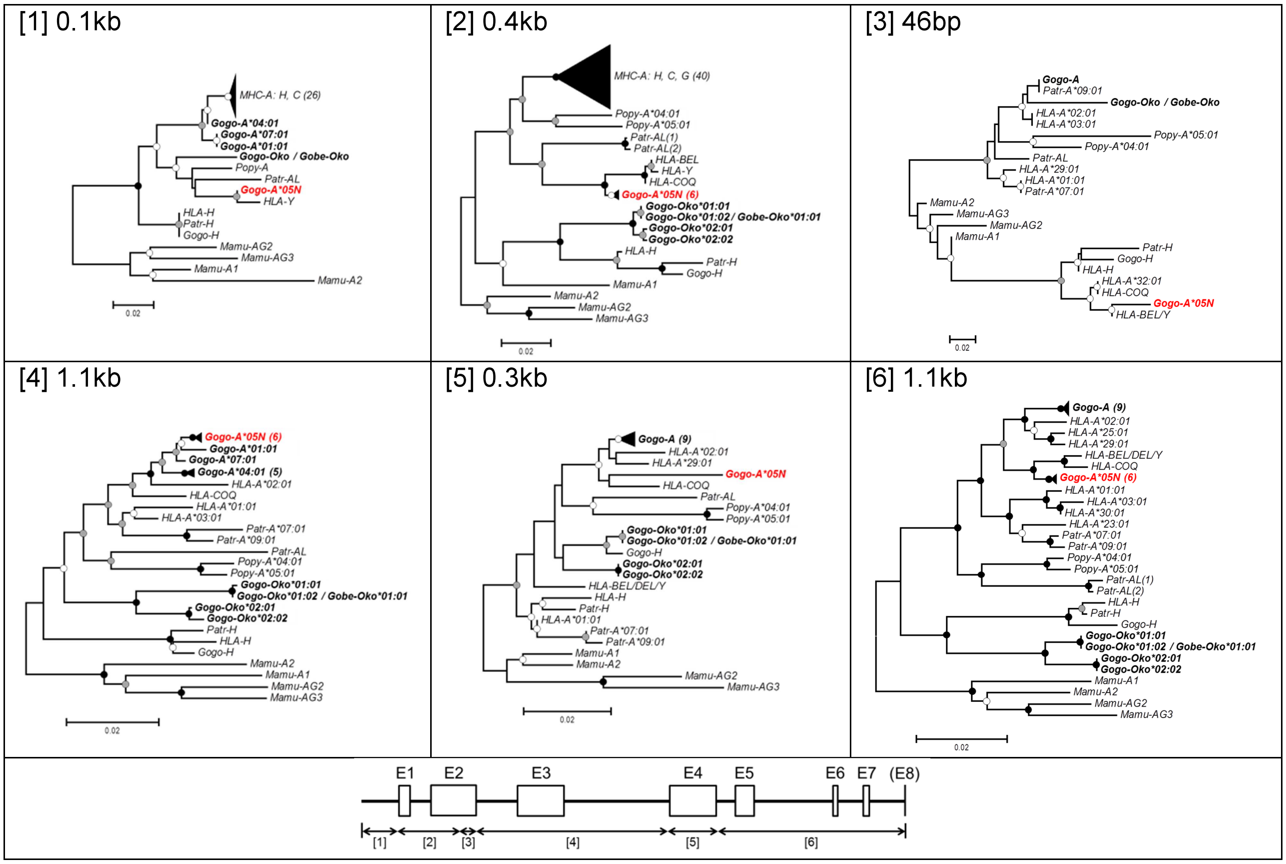

Supplement: Supplementary file 4 — (PDF 682 kb) [file 251_2017_974_MOESM4_ESM.pdf]
